# Expression and Function of Chemokines CXCL9-11 in Micturition Pathways in Cyclophosphamide (CYP)-Induced Cystitis and Somatic Sensitivity in Mice

**DOI:** 10.3389/fnsys.2018.00009

**Published:** 2018-04-06

**Authors:** Michael Guo, Phat Chang, Eric Hauke, Beatrice M. Girard, Katharine Tooke, Jacqueline Ojala, Susan M. Malley, Harrison Hsiang, Margaret A. Vizzard

**Affiliations:** Department of Neurological Sciences, The Robert Larner, M.D. College of Medicine, The University of Vermont, Burlington, VT, United States

**Keywords:** CXC chemokines, BPS/IC, ELISAs, cystometry, CXCR3, urinary bladder

## Abstract

Changes in urinary bladder function and somatic sensation may be mediated, in part, by inflammatory changes in the urinary bladder including the expression of chemokines. Male and female C57BL/6 mice were treated with cyclophosphamide (CYP; 75 mg/kg, 200 mg/kg, i.p.) to induce bladder inflammation (4 h, 48 h, chronic). We characterized the expression of CXC chemokines (CXCL9, CXCL10 and CXCL11) in the urinary bladder and determined the effects of blockade of their common receptor, CXCR3, at the level urinary bladder on bladder function and somatic (hindpaw and pelvic) sensation. qRT-PCR and Enzyme-Linked Immunoassays (ELISAs) were used to determine mRNA and protein expression of CXCL9, CXCL10 and CXCL11 in urothelium and detrusor. In urothelium of female mice treated with CYP, CXCL9 and CXCL10 mRNA significantly (*p* ≤ 0.01) increased with CYP treatment whereas CXC mRNA expression in the detrusor exhibited both increases and decreases in expression with CYP treatment. CXC mRNA expression urothelium and detrusor of male mice was more variable with both significant (*p* ≤ 0.01) increases and decreases in expression depending on the specific CXC chemokine and CYP treatment. CXCL9 and CXCL10 protein expression was significantly (*p* ≤ 0.01) increased in the urinary bladder with 4 h CYP treatment in female mice whereas CXC protein expression in the urinary bladder of male mice did not exhibit an overall change in expression. CXCR3 blockade with intravesical instillation of AMG487 (5 mg/kg) significantly (*p* ≤ 0.01) increased bladder capacity, reduced voiding frequency and reduced non-voiding contractions in female mice treated with CYP (4 h, 48 h). CXCR3 blockade also reduced (*p* ≤ 0.01) hindpaw and pelvic sensitivity in female mice treated with CYP (4 h, 48 h). CXC chemokines may be novel targets for treating urinary bladder dysfunction and somatic sensitization resulting from urinary bladder inflammation.

## Introduction

Bladder pain syndrome (BPS)/interstitial cystitis (IC) is one type of urologic chronic pelvic pain syndrome characterized by bladder-perceived pain, pressure or discomfort with at least one urinary symptom (Hanno and Sant, 2001). However, individuals with BPS/IC commonly report pain outside the pelvis. In the United States, many (2.7% to 6.5%) women have symptoms consistent with BPS/IC (Berry et al., 2011) but men (0.6% to 2.5%) are also diagnosed with BPS/IC (Clemens et al., 2014). The impact of BPS/IC on quality of life is enormous and the economic burden is significant (Clemens et al., 2014). Although the etiology and pathogenesis of BPS/IC are unknown, infection, inflammation, autoimmune disorder, toxic urinary agents, urothelial dysfunction and neurogenic causes have been proposed (Driscoll and Teichman, 2001; Sant and Hanno, 2001; Parsons, 2007; Patnaik et al., 2017). We have hypothesized that pain associated with BPS/IC involves a change in bladder sensory physiology. Altered urinary bladder sensations (i.e., pain at low or moderate bladder filling) with BPS/IC (Driscoll and Teichman, 2001; Sant and Hanno, 2001) may be mediated by multiple, possibly interacting, mechanisms including peripheral sensitization of bladder afferent pathways and an exaggerated response to normally innocuous stimuli (i.e., allodynia).

Potential mediators of urinary bladder inflammation and subsequent urinary bladder dysfunction and referred somatic sensitivity are numerous and include: chemokines (Sakthivel et al., 2008; Arms et al., 2010, 2013; Singh et al., 2013; Gonzalez et al., 2014a), cytokines (Malley and Vizzard, 2002; Yuridullah et al., 2006), neuropeptides (Vizzard, 2001; Braas et al., 2006), neuroactive compounds (Birder, 2005) and growth factors (Vizzard, 2000b; Yoshimura et al., 2006; Zvara and Vizzard, 2007). Chemokines, chemotactic cytokines, have well-established roles in the innate immune system, but are also nociceptive mediators and contribute to neuron-glia communication (Rutkowski and DeLeo, 2002; Watkins et al., 2003; Savarin-Vuaillat and Ransohoff, 2007; Milligan et al., 2008). Under physiologic conditions, chemokine expression is limited; however, following mechanical injury, infection or inflammation, chemokine expression increases in multiple cell types including neurons, glia, macrophages, T-cells and urothelial cells (Rutkowski and DeLeo, 2002; Tanaka et al., 2004; Verge et al., 2004; Lindia et al., 2005; White et al., 2005a,b; Bhangoo et al., 2007).

Our lab and others have previously demonstrated chemokine/receptor expression (CX3CL1/CX3CR1; CXCL12/CXCR4; Yuridullah et al., 2006; Vera et al., 2008; Arms et al., 2010, 2013; Gonzalez et al., 2014a) and function and referred somatic sensitivity (CXCL12/CXCR4; CCL2/CCR2; Tanaka et al., 2004; White et al., 2005a,b; Dansereau et al., 2008; Gosselin et al., 2008; Arms et al., 2010, 2013; Gonzalez et al., 2014a) associated with urinary bladder inflammation induced by cyclophosphamide (CYP) in rodents. CYP induces increased voiding frequency, and somatic sensitization (Guerios et al., 2008; Studeny et al., 2008; Cheppudira et al., 2009), and is associated with neurochemical (Vizzard, 2000b,c, 2001, 2006; Hu et al., 2005; Braas et al., 2006), organizational (Vizzard, 2000a) and electrophysiological (Yoshimura and de Groat, 1999; Yoshimura et al., 2002) plasticity in micturition pathways. Importantly, BPS/IC patients and patients with idiopathic overactive bladder (OAB) demonstrated increased serum expression of chemokines (Sakthivel et al., 2008; Tyagi et al., 2012; Furuta et al., 2018) and chemokine receptor binding (Ogawa et al., 2010). Furuta et al. (2018) have recently demonstrated increased expression of multiple chemokine markers in urine of IC patients with and without Hunner’s ulcer compared to OAB patients. OAB also has an elusive etiology but an inflammatory contribution has also been suggested (Steers, 2000; Compérat et al., 2006; Pillalamarri et al., 2017). Tyagi et al. (2012) detected an increase (10-fold) in the chemokine, CCL2, in the urine of OAB patients vs. controls.

The CXC chemokines are activated by infection and inflammation and exhibit pro-inflammatory (Murphy et al., 2014), anti-angiogenic (Mehrad et al., 2007) and nociceptive (White et al., 2005a,b) properties. CXCL9-11 and their common receptor, CXCR3, are thought to contribute to bladder inflammation in BPS/IC patients and preclinical murine models (Sakthivel et al., 2008; Singh et al., 2013) of urinary bladder inflammation. Serum levels of CXCL9-11 are elevated in both IC patients and CYP-induced murine models when compared to controls (Sakthivel et al., 2008). Several studies have addressed the contribution of CXC chemokines to urinary bladder inflammation induced by CYP (Sakthivel et al., 2008) or experimental autoimmune cystitis (EAC; Singh et al., 2013). In these studies, anti-CXCL10 antibody treatment ameliorated the severity of cystitis as determined with histological evaluation of urinary bladder and changes in leukocyte subpopulations and mast cells (Sakthivel et al., 2008; Singh et al., 2013). In the present study using male and female C57BL/6 mice with CYP-induced cystitis (4 h, 48 h, chronic), we extend these observations and examine: (1) CXC chemokines (CXCL9-11) mRNA and protein expression in the urinary bladder (urothelium, detrusor smooth muscle); (2) effects of CXCR3 receptor blockade on urinary bladder function and somatic sensitivity (hindpaw and pelvis) using conscious cystometry and von Frey filament testing, respectively; and (3) mRNA and protein expression of CXCR3 in the urinary bladder.

## Materials and Methods

### Animals

Male and female C57BL/6 wildtype (WT) mice used in this study were bred locally at the Larner College of Medicine at The University of Vermont. The litters were of normal size, weight, and activity (feeding, drinking, behaviors). The UVM Institutional Animal Care and Use Committee approved all experimental protocols involving animal use (IACUC # 08-055). Animal care was under the supervision of the UVM Office of Animal Care Management in accordance with the Association for Assessment and Accreditation of Laboratory Animal Care (AAALAC) and National Institutes of Health (NIH) guidelines. Estrous cycle status was not determined in female mice before use. All efforts were made to minimize the potential for animal pain, stress, or distress. Separate groups of littermate WT mice were used in the following experiments.

### CYP-Induced Cystitis and Tissue Harvest for mRNA and Protein Studies

Male (*n* = 6–7, per treatment group) and female adult (3–4 months) mice (*n* = 6–7, per treatment group) received CYP intraperitoneally to create 4 h, 48 h (200 mg/kg) and chronic (75 mg/kg; every third for a total of three injections) treatment groups (Vizzard, 2000a, 2006; Braas et al., 2006; Gonzalez et al., 2013). The control group received no CYP treatments. Mice were deeply anesthetized with isoflurane (5% in oxygen), euthanized via thoracotomy and bladders were harvested from all CYP treatment groups. The urinary bladder was quickly dissected under RNase-free conditions. For mRNA analysis, the bladder was cut open along the midline and pinned to a sylgard-coated dish and the urothelium (which includes the suburothelium) was removed from the detrusor muscle with fine forceps and iris scissors. The whole urinary bladder from each treatment group (*n* = 6–8, per treatment group for each sex) was used for protein analyses. Separate groups of male and female mice were used for RNA and protein analyses.

### Split Bladder Preparation and Assessment of Potential Contamination of Bladder Layers

The urothelium + suburothelium was dissected from the detrusor smooth muscle using fine forceps under a dissecting microscope as previously described (Corrow et al., 2010; Schnegelsberg et al., 2010). To confirm the specificity of our split bladder preparations, urothelium + suburothelium and detrusor samples were examined for the presence of α-smooth muscle actin and uroplakin II as previously described (Corrow et al., 2010; Girard et al., 2011, 2013). In these studies, the use of the term urothelium refers to the urothelium and suburothelial layers.

### Quantitative RT-PCR for mRNA Quantification

Quantitative RT-PCR (Girard et al., 2008, 2010, 2013, 2016; Gonzalez et al., 2013) was used to determine CXCL9, CXCL10, and CXCL11 mRNA transcript levels in the urothelium and detrusor muscle as previously described. Total RNA was extracted using STAT-60 total RNA/mRNA isolation (Tel-Test “B”, Friendswood, TX, USA). One microgram of RNA per sample was used to synthesize complementary DNA using a mix of random hexamer and oligo dT primers with M-MLV reverse transcriptase (Promega Corp., Madison, WI, USA) in a 25-μL final reaction volume. The quantitative PCR standards for all transcripts were prepared with the amplified cDNA products ligated directly into pCR2.1 TOPO vector using the TOPO TA cloning kit (Invitrogen, Carlsbad, CA, USA). The nucleotide sequences of the inserts were verified by automated fluorescent dideoxy dye terminator sequencing (Vermont Cancer Center DNA Analysis Facility). To estimate the relative expression of the receptor transcripts, 10-fold serial dilutions of stock plasmids were prepared as quantitative standards. The range of standard concentrations was determined empirically. Complementary DNA templates, diluted 10-fold to minimize the inhibitory effects of the reverse transcription reaction components, were assayed using HotStart-IT SYBR Green qPCR Master Mix (USB, Cleveland, OH, USA) and 300 nM of each primer in a final 25-μL reaction volume. Real-time quantitative PCR was performed on an Applied Biosystems 7500 Fast real-time PCR system (Applied Biosystems, Foster City, CA, USA) as previously described (Girard et al., 2008, 2010, 2013, 2016; Gonzalez et al., 2013). Oligonucleotide primer sequences were: CXCL9: upper primer (5′-GTGGAGTTCGAGGAACCCTAG-3′); lower primer (5′-ATTGGGGCTTGGGGCAAAC-3′); CXCL10: upper primer (5′-GTGGGACTCAAGGGATCCCTC-3′); lower primer (5′-CAGGATAGGCTCGCAGGGATG-3′); CXCL11: upper primer (5′-GCTCAAGGCTTCCTTATGTTCAAAC-3′); lower primer (5′-CTTTGTCGCAGCCGTTACTCG-3′). Commercially available oligonucleotide primers for CXCR3 were purchased from Applied Biosystems (ThermoFisher Scientific, Springfield, NJ, USA; TaqMan TM Gene Expression Assay, catalog number 4453320).

### Data Analysis for mRNA Quantification

For data analyses, a standard curve was constructed by amplification of serially diluted plasmids containing the target sequence. Data were analyzed at the termination of each assay using sequence detection software (Sequence Detection Software, version 1.3.1; Applied Biosystems, Norwalk, CT, USA; Girard et al., 2008, 2010, 2013, 2016; Gonzalez et al., 2013) as previously described. Data are expressed as the relative quantity of the gene of interest normalized to the relative quantity of the housekeeping gene, ribosomal protein L32.

### Enzyme-Linked Immunoassays (ELISAs) for Protein Quantification

ELISAs were used to determine CXCL9, CXCL10 and CXCL11 protein content in the urinary bladder of mice (n = 6–8) with and without CYP treatment as previously described (Vizzard, 2000b; Schnegelsberg et al., 2010; Gonzalez et al., 2015). Protein samples from male and female mice were assayed simultaneously on the same ELISA plates to enable quantitative comparison between male and female samples. According to the manufacturer (R&D Systems, Minneapolis, MN, USA), the CXCL9, CXCL10 and CXCL11 DuoSets do not show cross-reactivity or interference with similar recombinant human and mouse proteins at concentrations up to 50 ng/ml. The standards provided with these systems generated linear standard curves (*R*^2^ = 0.985–0.998, *p* ≤ 0.001). No samples fell below the detection limits of the assays, and samples were not diluted before assay. Curve fitting of standards and evaluation of chemokine content of samples was performed using a least squares fit as previously described (Vizzard, 2000b; Schnegelsberg et al., 2010; Gonzalez et al., 2015).

### Urinary Bladder Wholemount Preparation and Cryostat Sections, Immunohistochemistry and Image Analyses

CXCR3-immunoreactivity (IR) was evaluated in urinary bladder wholemount preparations and cryostat sections (full tissue thickness). The bladder (*n* = 5 from each treatment group) was cut open along the midline and pinned to a Sylgard-coated dish. Notches were made on one side of the bladder neck for orientation purposes. The bladder was incubated for 1.5 h at room temperature in cold fixative (2% paraformaldehyde + 0.2% picric acid). Fine-tipped forceps and iris scissors were used to dissect the urothelium + suburothelium from the underlying detrusor smooth muscle with the aid of a dissecting microscope (Arms et al., 2010, 2013; Schnegelsberg et al., 2010; Girard et al., 2016). Cryostat sections (20 μm) from whole urinary bladder were also used to evaluate CXCR3-IR in cross-sections of urinary bladder from treatment groups (*n* = 3) using an on-slide processing technique. Tissues were blocked with PBS (pH 7.4) containing 20% normal goat serum, 0.2% Triton X-100 for 2 h at room temperature and the primary antibody (rabbit anti-CXCR3, 1:1K; Sigma, St. Louis, MO, USA) was applied in PBS containing 4% normal goat serum, 0.2% Triton X-100 overnight at 4°C (Arms et al., 2010, 2013). Tissues were washed in PBS (pH 7.4) containing 0.1% BSA, 0.1% Triton-X-100, 4× for 15 min each at room temperature. The secondary antibody (Cyanine (Cy3)-conjugated goat anti-rabbit antibody (1:500; Jackson ImmunoResearch, West Grove, PA, USA) was applied and incubated for 2 h at room temperature in the same solution as the primary antibody. The tissue was washed 3× for 10 min each in PBS and mounted with mounting medium (Polysciences, Warrington, PA, USA). In some wholemount preparations processed for CXCR3-IR, nerve fibers in the suburothelial nerve plexus were also stained with the pan-neuronal marker protein gene product (PGP9.5, 1:3K; AbD Serotec, Raleigh, NC, USA) to determine potential expression of CXCR3 in suburothelial nerve fibers and visualized with Cy2-conjugated species-specific secondary antibodies (1:200; Jackson ImmunoResearch Laboratories).

### Assessment of Immunohistochemical Staining in Urinary Bladder

Tissues from WT and treatment groups were examined for CXCR3-IR simultaneously using an Olympus fluorescence photomicroscope (Olympus Corporation of the Americas, Center Valley, PA, USA; Arms et al., 2010, 2013; Girard et al., 2016). Cy3 was visualized with a filter with an excitation range of 560–596 nm and an emission range of 610–655 nm. Methodological and procedural controls were performed. CXCR3-IR that was greater than the background level in experiment-matched negative controls was considered positively stained.

### Figure Preparation

Digital images were obtained using a charge-coupled device camera (MagnaFire SP, Optronics, Optical Analysis, Nashua, NH, USA) and LG-3 frame grabber attached to an Olympus microscope (Arms et al., 2010, 2013; Girard et al., 2016). Images were imported into Photoshop 7.0 (Adobe Systems, San Jose, CA, USA), where groups of images were assembled and labeled.

### Intravesical Catheter Implant

Female mice (*n* = 8–10 were anesthetized with isoflurane in oxygen (3%–4%) and administered analgesics preoperatively (carprofen, 0.1 mg/kg, s.c.). A lower midline abdominal incision was made, and polyethylene tubing (PE-10, Clay Adams, Parsippany, NJ, USA) was inserted into the bladder dome and secured with nylon purse-string sutures (6-zero) as previously described (Schnegelsberg et al., 2010; Gonzalez et al., 2013). Mice received postoperative analgesics (carprofen, 0.1 mg/kg, s.c.) every 24 h for a total of 48 h after surgery.

### Conscious, Open Outlet, Continuous Fill Cystometry

For cystometry in conscious mice (control, 4 h, 48 h), an unrestrained animal was placed in a Plexiglass cage with a wire bottom, the bladder was emptied and the catheter was connected via a T-tube to a pressure transducer (Grass Model PT300, West Warwick, RI, USA) and microinjection pump (Harvard Apparatus 22, South Natick, MA, USA). A Small Animal Cystometry Lab Station (MED Associates, St. Albans, VT, USA) was used for urodynamic measurements (Schnegelsberg et al., 2010; Gonzalez et al., 2013) as previously described. Saline solution was infused at room temperature into the bladder at a rate of 25 μl/min to elicit repetitive bladder contractions. At least six reproducible micturition cycles were recorded after the initial stabilization period of 25–30 min (Schnegelsberg et al., 2010; Gonzalez et al., 2013). The following cystometric parameters were recorded in each animal: minimum pressure (pressure at the beginning of the bladder filling), threshold pressure (bladder pressure immediately prior to micturition), maximum micturition pressure, intercontraction interval (time between micturition events), infused volume, void volume, presence and amplitude of non-voiding bladder contractions (NVCs; Schnegelsberg et al., 2010; Gonzalez et al., 2013). NVCs were defined as rhythmic intravesical pressure increases 5 cm H_2_0 above baseline, during the filling phase, without the release of fluid from the urethra. Infused volume was measured as the volume of saline infused into the bladder at the time when micturition commenced (Schnegelsberg et al., 2010; Gonzalez et al., 2013). Mice in these studies had residual volume of less than 5 μl; therefore, infused volume and void volume were similar. At the conclusion of the experiment, mice were euthanized (5% isoflurane plus thoracotomy).

### Conscious Cystometry and Effects of AMG487, a Potent and Selective CXCR3 Antagonist

The effects of AMG487 (5 mg/kg), a potent and selective CXCR3 antagonist (Walser et al., 2006; Guan et al., 2015), on urinary bladder function in littermate WT mice (control, 4 h, 48 h) were assessed using conscious, open outlet, cystometry with continuous instillation of intravesical saline (0.9%; Schnegelsberg et al., 2010; Gonzalez et al., 2013). For intravesical administration of AMG487, mice were anesthetized with 2% isoflurane and AMG487 (5 mg/kg; <300 μl) was injected through the bladder catheter; the animals were maintained under anesthesia to prevent expulsion of AMG487 via a voiding reflex. In this procedure, AMG487 remained in the bladder for 30 min at which time, the drug was drained, the bladder washed with saline (0.9%) and animals recovered from anesthesia for 20 min before experimentation. Cystometry experiments were performed before and after vehicle or AMG487 intravesical instillation. The concentration (5 mg/kg) of AMG487 used in these studies was based upon previous (Walser et al., 2006; Guan et al., 2015) and pilot studies. At the conclusion of the experiment, mice were euthanized (4%–5% isoflurane plus thoracotomy). Experiments were conducted at similar times of the day to avoid the possibility that circadian variations were responsible for changes in cystometric measurements. Individuals blinded to genotype analyzed the cystometric data; groups were decoded after data analysis (Schnegelsberg et al., 2010; Gonzalez et al., 2013).

### Mechanical Sensitivity Testing

Referred (secondary) hyperalgesia was measured by testing the frequency of withdrawal responses to the application of calibrated von Frey monofilaments to the abdomen (Cheppudira et al., 2009; Schnegelsberg et al., 2010) region overlying the urinary bladder with CXCR3 antagonist, AMG487 (5 mg/kg) delivered intravesically via a transurethral catheter to avoid the need for an abdominal incision. In this procedure, AMG487 (5 mg/kg; <300 μl) remained in the bladder for 30 min at which time, the drug was drained, the bladder washed with saline, the catheter removed and animals recovered from anesthesia for 20 min before experimentation. Mechanical sensitivity assessment was performed using von Frey monofilaments (Stoelting, Wood Dale, IL, USA) with forces of 0.1–4 g applied to the pelvic region or hindpaw (Cheppudira et al., 2009; Schnegelsberg et al., 2010). All mice were first habituated in a clear acrylic testing chamber 20 min/day for 4 days with a fan to generate ambient noise. On day of testing, the mice were placed in the acrylic testing chamber on top of a metal mesh floor (IITC Life Science Inc., Woodland Hills, CA, USA) and habituated again for 10 min before the application of von Frey filaments in an up-down method for 1–3 s with a minimum interstimulus interval of 2 min (Cheppudira et al., 2009; Schnegelsberg et al., 2010). For pelvic region stimulation, stimulation was confined to the lower abdominal area overlying the urinary bladder. Positive responses to hindpaw or pelvic region stimulation have been previously described (Cheppudira et al., 2009; Schnegelsberg et al., 2010). Somatic sensitivity before and after vehicle or AMG487 intravesical instillation was evaluated in the same mice (control and 4 h CYP-induced cystitis; *n* = 10 each). Separate groups of mice were used for cystometry and somatic sensitivity testing. All somatic testing was performed in a blinded manner with respect to treatment. The groups were decoded after data analysis.

### AMG487 Preparation

AMG487 was purchased from ThermoFisher Scientific, Springfield, NJ, USA. AMG487 was used intravesically at 5 mg/kg (Walser et al., 2006; Guan et al., 2015). Briefly, a 50% hydroxypropyl-β-cyclodextrin (Sigma, St. Louis, MO, USA) solution was prepared; at 20%, this solution served as the vehicle. AMG487 was added to the 50% solution and incubated in a sonicating water bath for 2 h with vortexing. Distilled water was added to achieve the final concentration of AMG487 in 20% of hydroxypropyl-β-cyclodextrin.

### Exclusion Criteria

Several mice of both sexes (*n* = 4 total) were removed due to postoperative lethargy attributed to dehydration that was not successfully managed. In addition, behavioral movements such as grooming and defecation rendered bladder pressure recordings during these events unusable.

### Statistical Analyses

All values are means ± SEM. Comparisons among experimental groups were made using analysis of variance (ANOVA) or repeated measures ANOVA (cystometry and somatic sensitivity data). Animals, processed and analyzed on the same day, were tested as a block in the ANOVA. When *F* ratios exceeded the critical value (*p* ≤ 0.05), the Tukey’s (HSD) *post hoc* test was used to compare means among groups.

## Results

### CXC mRNA Transcript Levels With and Without CYP-Induced Cystitis in Urothelium and Detrusor of Female Mice

#### Urothelium

**Figure 1:**
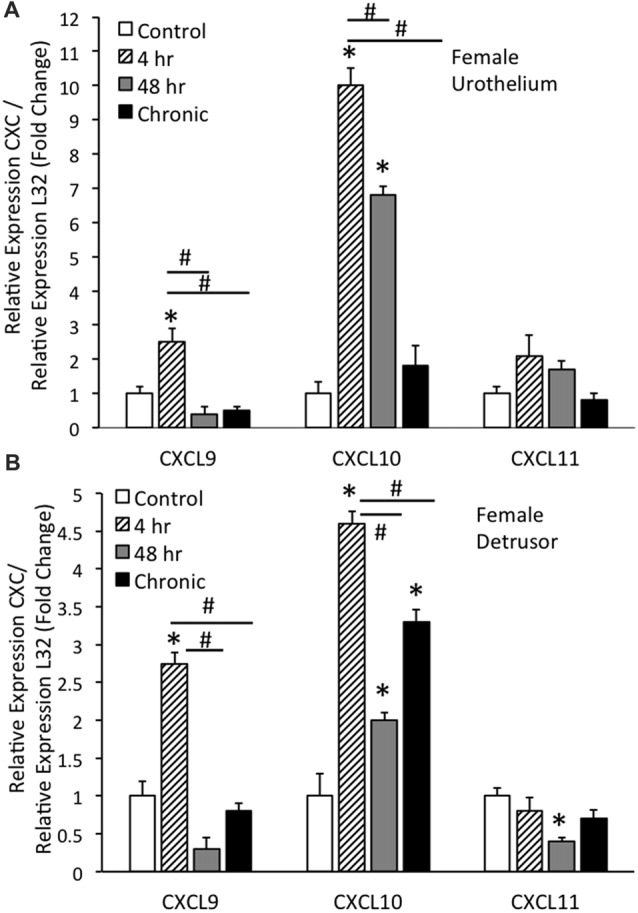
CXC mRNA transcript expression in female mouse urothelium **(A)** and detrusor **(B)** and the effects of cyclophosphamide (CYP)-induced cystitis (4 h, 48 h, chronic) as determined by quantitative PCR (qRT-PCR). **(A)** CXCL9 and CXCL10 mRNA levels increased significantly (*p ≤ 0.01) in the urothelium with 4 h CYP treatment compared to control. CXCL10 mRNA expression also increased significantly (*p ≤ 0.01) in the urothelium with 48 h CYP treatment compared to control. CXCL9 and CXCL10 mRNA expression with acute 4 h CYP-induced cystitis was significantly (^#^*p* ≤ 0.01) greater than that observed with 48 h and chronic CYP treatment. No changes in CXCL11 mRNA expression were demonstrated in the urothelium with CYP treatment of any duration. **(B)** CXCL9 and CXCL10 mRNA levels were significantly (*p ≤ 0.01) increased in the detrusor of female mice with 4 h CYP treatment compared to control and these levels were significantly (^#^*p* ≤ 0.01) greater than those detected at the 48 h and chronic time points. CXCL10 mRNA expression in the detrusor of female mice was significantly (*p ≤ 0.01) increased with all CYP treatment durations compared to control. CXCL11 mRNA expression was significantly (*p ≤ 0.01) decreased in the detrusor of female mice with 48 h CYP treatment compared to control. CXC mRNA is expressed as the fold change of relative CXC expression/relative L32 expression and normalized to expression in urothelium **(A)** or detrusor **(B)** from control mice. Sample sizes are *n* = 6–7 for each group.

Real-time qRT-PCR experiments demonstrated significant (*p* ≤ 0.01) increases in CXCL9 mRNA transcript expression in the urothelium of female mice with acute (4 h) CYP treatment (Figure 1A). CXCL9 transcript expression in urothelium of female mice with acute (4 h) CYP treatment was significantly (*p* ≤ 0.01) greater than that with intermediate (48 h) and chronic CYP treatment (Figure 1A). qRT-PCR also demonstrated significant (*p* ≤ 0.01) increases in CXCL10 mRNA transcript expression in the urothelium of female mice with acute (4 h) and intermediate (48 h) CYP-induced cystitis (Figure 1A). CXCL10 mRNA expression in female urothelium with acute (4 h) cystitis was significantly (*p* ≤ 0.01) greater than that with intermediate (48 h) and chronic CYP-induced cystitis (Figure 1A). In contrast, no changes in CXCL11 transcript expression were demonstrated in urothelium of female mice with any duration of CYP treatment (Figure 1A).

#### Detrusor

Real-time qRT-PCR experiments demonstrated significant (*p* ≤ 0.01) increases in CXCL9 mRNA transcript expression in the detrusor of female mice with acute (4 h) CYP treatment (Figure 1B). CXCL9 transcript expression in detrusor of female mice with acute (4 h) CYP treatment was significantly (*p* ≤ 0.01) greater than that with intermediate (48 h) and chronic CYP treatment (Figure 1B). qRT-PCR also demonstrated significant (*p* ≤ 0.01) increases in CXCL10 mRNA transcript expression in the detrusor of female mice with acute (4 h), intermediate (48 h) and chronic CYP-induced cystitis (Figure 1B). CXCL10 mRNA expression in female detrusor with acute (4 h) cystitis was significantly (*p* ≤ 0.01) greater than that with intermediate (48 h) and chronic CYP-induced cystitis (Figure 1B). Intermediate (48 h) CYP-induced cystitis significantly (*p* ≤ 0.01) decreased CXCL11 transcript expression in detrusor of female mice (Figure 1B).

### CXC mRNA Transcript Levels With and Without CYP-Induced Cystitis in Urothelium and Detrusor of Male Mice

#### Urothelium

**Figure 2:**
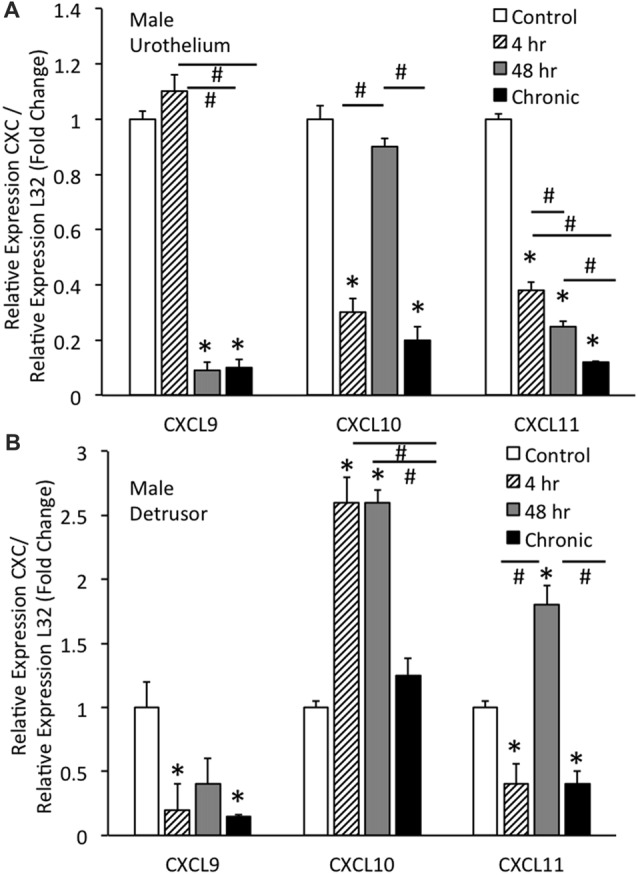
CXC mRNA transcript expression in male mouse urothelium **(A)** and detrusor **(B)** and the effects of CYP-induced cystitis (4 h, 48 h, chronic) as determined by quantitative PCR (qRT-PCR). **(A)** CXCL9 and CXCL11 mRNA levels decreased significantly (*p ≤ 0.01) in the urothelium with 48 h and chronic CYP treatment compared to control and CXCL11 mRNA levels also significantly (*p ≤ 0.01) decreased in the urothelium with 4 h CYP treatment compared to control. CXCL10 mRNA expression also decreased significantly (*p ≤ 0.01) in the urothelium with 4 h and chronic CYP treatment compared to control. CXCL9 mRNA expression was significantly (^#^*p* ≤ 0.01) decreased with 48 h and chronic CYP treatment compared to 4 h CYP treatment. CXCL11 mRNA expression was progressively and significantly (*p ≤ 0.01) decreased with increasing duration of CYP treatment (4 h > 48 h > chronic CYP). **(B)** CXCL9 and CXCL11 mRNA levels were significantly (*p ≤ 0.01) decreased in the detrusor of male mice with 4 h CYP and chronic CYP treatment compared to control. CXCL10 mRNA expression in the detrusor of female mice was significantly (*p ≤ 0.01) increased with 4 h and 48 h CYP treatment durations compared to control (*) and chronic CYP treatment (#). CXCL11 mRNA expression was significantly (*p ≤ 0.01) increased in the detrusor of male mice with 48 h CYP treatment compared to control and this expression was significantly (^#^*p* ≤ 0.01) greater than CXCL11 mRNA expression at 4 h and chronic CYP treatment. CXC mRNA is expressed as the fold change of relative CXC expression/relative L32 expression and normalized to expression in urothelium **(A)** or detrusor **(B)** from control mice. Sample sizes are *n* = 6–7 for each group.

Real-time qRT-PCR experiments demonstrated significant (*p* ≤ 0.01) decreases in CXCL9 mRNA transcript expression in the urothelium of male mice with intermediate (48 h) CYP and chronic CYP treatment (Figure 2A). CXCL9 transcript expression in urothelium of male mice with intermediate (48 h) and chronic CYP treatment was significantly (*p* ≤ 0.01) decreased compared to acute (4 h) CYP-induced cystitis (Figure 2A). qRT-PCR also demonstrated significant (*p* ≤ 0.01) decreases in CXCL10 mRNA transcript expression in the urothelium of male mice with acute (4 h) and chronic CYP-induced cystitis (Figure 2A). CXCL10 mRNA expression in male urothelium with acute (4 h) and chronic cystitis was significantly (*p* ≤ 0.01) decreased compared to intermediate (48 h) CYP-induced cystitis (Figure 2A). CXCL11 transcript expression in male urothelium was significantly (*p* ≤ 0.01) decreased with all durations of CYP treatment. CXCL11 mRNA expression in male urothelium with acute (4 h) cystitis was significantly (*p* ≤ 0.01) greater than expression with 48 h or chronic CYP treatment (Figure 2A). CXCL11 mRNA expression in male urothelium with intermediate (48 h) CYP-induced cystitis was significantly (*p* ≤ 0.01) greater than expression with chronic CYP treatment (Figure 2A).

#### Detrusor

Real-time qRT-PCR experiments demonstrated significant (*p* ≤ 0.01) decreases in CXCL9 mRNA transcript expression in the detrusor of male mice with acute (4 h) and chronic CYP treatment (Figure 2B). qRT-PCR demonstrated significant (*p* ≤ 0.01) increases in CXCL10 mRNA transcript expression in the detrusor of male mice with acute (4 h) and intermediate (48 h) CYP-induced cystitis (Figure 2B). CXCL10 mRNA expression in male detrusor with acute (4 h) cystitis and intermediate (48 h) cystitis was significantly greater than that following chronic CYP-induced cystitis (Figure 2B). Intermediate (48 h) CYP-induced cystitis significantly (*p* ≤ 0.01) increased CXCL11 transcript expression in detrusor of male mice (Figure 2B) whereas 4 h and chronic CYP-induced cystitis significantly (*p* ≤ 0.01) reduced CXCL11 mRNA transcript expression (Figure 2B). CXCL11 mRNA expression in male detrusor with intermediate (48 h) cystitis was significantly (*p* ≤ 0.01) greater than that following acute (4 h) and chronic CYP-induced cystitis (Figure 2B).

### CXC Protein Expression in the Whole Urinary Bladder With CYP-Induced Cystitis in Female and Male Mice

#### Female

**Figure 3:**
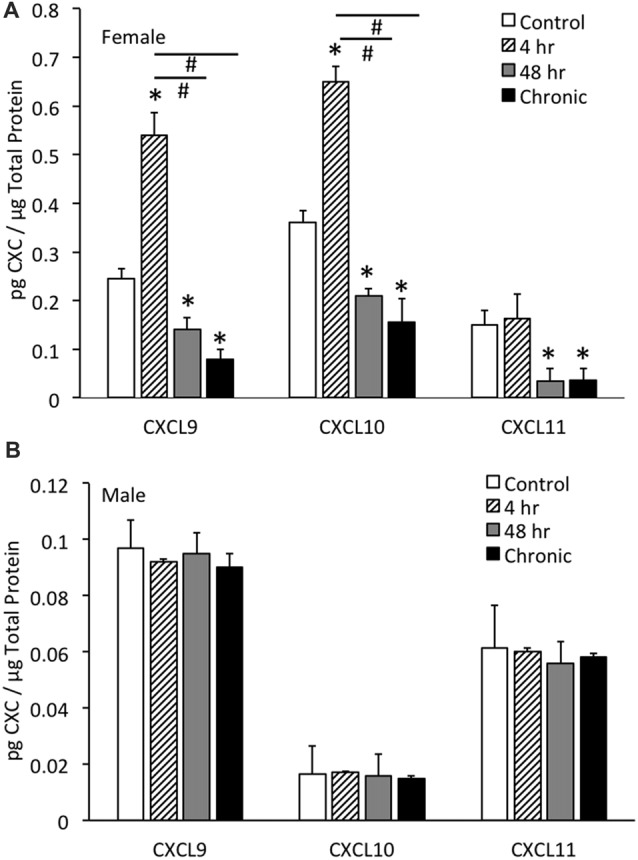
CXC chemokine (CXCL9, CXCL10, CXCL11) protein expression in whole urinary bladders of female **(A)** and male **(B)** mice following CYP treatment of varying duration (4 h, 4 h, 48 h, chronic) as determined with enzyme-linked immunosorbent assays. CXCL9 and CXCL10 chemokine protein expression increased significantly (*p ≤ 0.01) in whole urinary bladder following 4 h CYP treatment in female mice **(A)** compared to control (*p ≤ 0.01) and 48 h and chronic treatments (^#^*p* ≤ 0.01). CXCL9, CXCL10 and CXCL11 protein expression in urinary bladder was significantly (*p ≤ 0.01) decreased with 48 h and chronic CYP treatment compared to control females **(A)**. Sample sizes are *n* = 6–8 for each group. CXC (CXCL9, CXCL10, CXCL11) protein expression in whole urinary bladders of male mice was not regulated following CYP-induced cystitis of any duration evaluated **(B)**. CXC protein (CXCL9, CXCL10, CXCL11) expression in female whole urinary bladder was significantly (*p* ≤ 0.01) greater than expression in male urinary bladder under control conditions **(A,B)**. Sample sizes are *n* = 6–8 for each group. ELISAs for CXC chemokines in urinary bladder of male mice were repeated with an additional *n* = 6–8 mice per group.

CXCL9 protein expression in the whole urinary bladder of female mice increased significantly (*p* ≤ 0.01) with all durations of CYP treatment evaluated compared to control urinary bladders (no CYP) as determined with ELISAs (Figure 3A). CXCL9 protein expression in whole urinary bladder of female mice with 4 h CYP-induced cystitis was significantly greater than that observed following intermediate (48 h) and chronic CYP treatment. The magnitude and pattern of changes in CXCL10 protein expression in the whole urinary bladder of female mice treated with CYP was comparable to that demonstrated for CXCL9 (Figure 3A). In contrast, CXCL11 protein expression in the whole urinary bladder of female mice significantly (*p* ≤ 0.01) decreased with intermediate (48 h) and chronic CYP-induced cystitis (Figure 3A).

#### Male

No changes in CXCL9, CXCL10 or CXCL11 protein expression in the whole urinary bladder of male mice was observed with any duration of CYP treatment (Figure 3B). CXC protein (CXCL9, CXCL10, CXCL11) expression in female whole urinary bladder was significantly (*p* ≤ 0.01) greater than expression in male urinary bladder under control conditions (Figures 3A,B).

In contrast to the regulation of CXC protein expression in whole urinary bladder in female mice with CYP-induced cystitis (Figure 3A), CXC protein expression was not regulated at the level of the whole urinary bladder in male mice with CYP-induced cystitis (Figure 3B). In addition, CXC protein expression in male mice was significantly (*p* ≤ 0.01) less than CXC protein expression in whole urinary bladder from female mice (Figures 3A,B). Thus, the following studies examining the effects of a CXCR3 receptor antagonist on bladder function and somatic sensitivity have used female mice.

### Effects of CXCR3 Receptor Blockade With Intravesical Infusion of AMG487 Using Conscious Cystometry in Female Mice With or Without CYP-Induced Cystitis (4 h, 48 h)

#### Control (No Inflammation)

**Figure 4:**
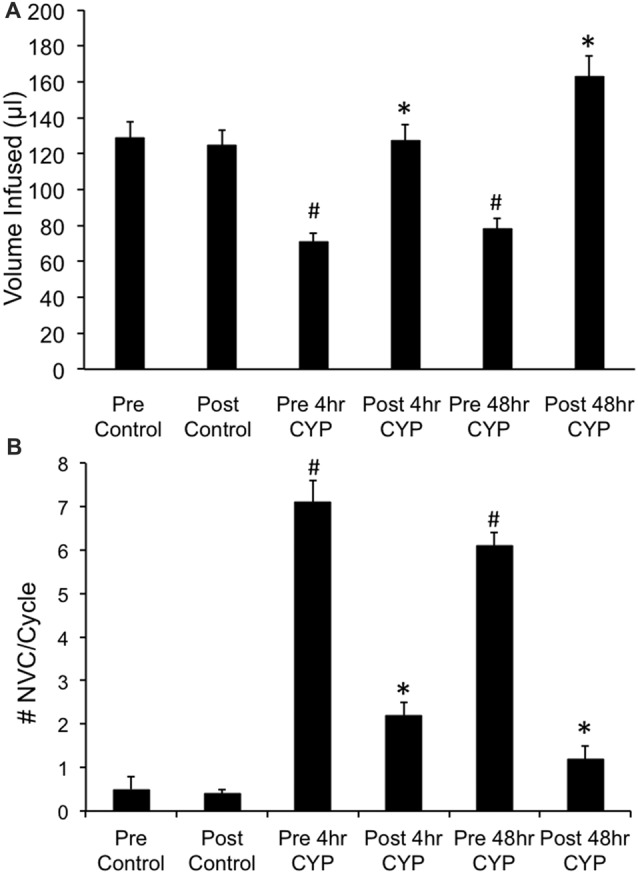
Summary histograms of the effects of intravesical instillation of a CXCR3 receptor antagonist (AMG487; 5 mg/kg) on bladder capacity defined as infused volume (μl) **(A)** to elicit a micturition reflex and the number (#) of non-voiding contractions (NVCs/micturition cycle) **(B)** (increases in bladder pressure of ≥5 cm H_2_0 during the filling phase without the release of urine) in control and CYP-treated (4 h, 48 h) female mice. Bladder function testing was performed before (pre) and after (post) intravesical administration of a CXCR3 receptor antagonist in the same groups of control, 4 h and 48 h CYP-treated mice. Intravesical instillation of AMG487 (5 mg/kg) significantly (*p* ≤ 0.01) increased volume infused (μl) **(A)**, and significantly (*p* ≤ 0.01) decreased the # of NVCs/cycle **(B)** in 4 h and 48 h CYP-treated female mice. *p ≤ 0.01 compared to pre AMG487 treatment. ^#^*p* ≤ 0.01 compared to control (before or after AMG487 treatment). Sample sizes are *n* = 8–10 in each group.

**Figure 5:**
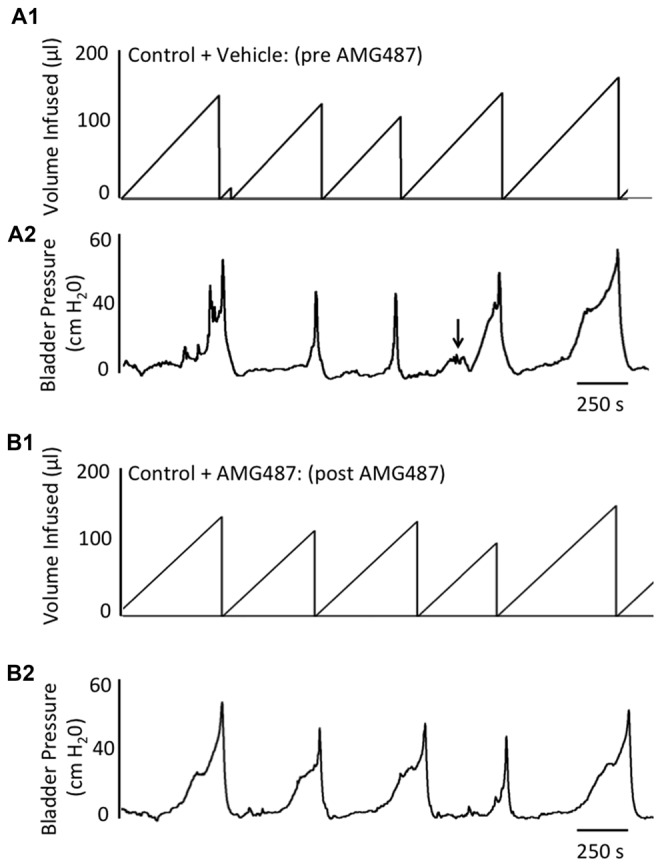
Representative cystometrogram recordings using continuous intravesical infusion of saline in conscious, female mice with an open outlet from a control (no inflammation) mouse before (pre **A1**,**A2**) and after (post **B1**,**B2**) intravesical CXCR3 receptor blockade with AMG487 (5 mg/kg). **(A)** Volume infused (μl; **A1**,**B1**) and bladder pressure (cm H_2_0; **A2**,**B2**) before (vehicle) **(A1, A2)** and after AMG487 (5 mg/kg) instillation **(B1,B2)** in a control female mouse. Bladder function recordings in **(A1,A2)** and **(B1,B2)** are from the same mouse. Arrow **(A2)** indicates appearance of a non-voiding contraction (≥5 cm H_2_0 without the release of urine during the filling phase).

Conscious cystometry was performed in control female mice with vehicle before intravesical infusion (pre control) of AMG487 (5 mg/kg), a CXCR3 receptor antagonist, to establish baseline voiding frequency, bladder capacity (Figures 4A, 5A1,A2), non-voiding contractions (presence and amplitude; Figures 4B, 5A1,A2) and void volume (data not shown). Drug treatment with AMG487 (post control) did not change bladder capacity, as measured as the amount of saline infused in the bladder at the time when micturition commenced (Figures 4A, 5B1,B2), void frequency, non-voiding contractions (presence of amplitude; Figures 4B, 5B1,B2) or threshold, minimum, average or maximum micturition pressure in control female mice (Figure 7).

**Figure 6:**
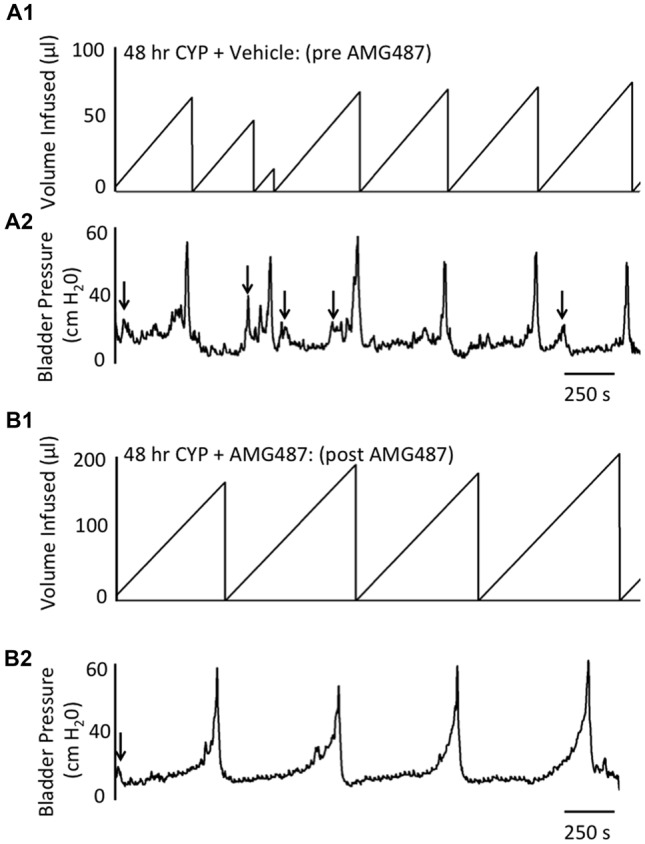
Representative cystometrogram recordings using continuous intravesical infusion of saline in conscious, female mice with an open outlet following CYP-treatment (48 h) before (pre **A1**,**A2**) and after (post **B1**,**B2**) intravesical CXCR3 receptor blockade with AMG487 (5 mg/kg). **(A)** Volume infused (μl; **A1**,**B1**) and bladder pressure (cm H_2_0; **A2**,**B2**) before (vehicle; **A1**,**A2**) and after AMG487 (5 mg/kg) instillation **(B1,B2)** in a female mouse treated with CYP 48 h. Bladder function recordings in **(A1,A2)** and **(B1,B2)** are from the same mouse. Arrows** (A2,B2)** indicate appearance of non-voiding contractions (≥5 cm H_2_0 without the release of urine during the filling phase).

**Figure 7:**
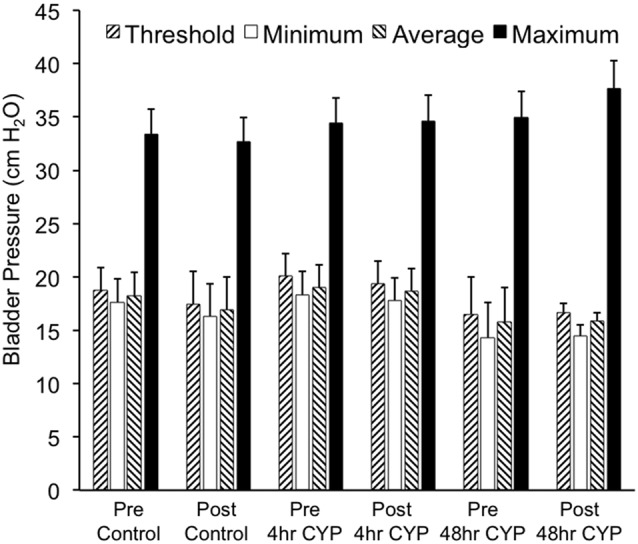
Summary histogram of the effects of intravesical instillation of a CXCR3 receptor antagonist (AMG487; 5 mg/kg) on bladder pressures (cm H_2_O; threshold, minimum, average and maximum) in control and CYP-treated (4 h, 48 h) female mice. Bladder function testing and recording of bladder pressures was performed before (pre) and after (post) intravesical administration of a CXCR3 receptor antagonist in the same groups of control, 4 h and 48 h CYP-treated mice. No changes in bladder pressures were observed. Sample sizes are *n* = 8–10 for each group.

#### 4 h CYP Treatment

Conscious cystometry was performed in female mice treated with CYP treatment (4 h) and vehicle before intravesical infusion of AMG487 (5 mg/kg; pre 4 h CYP) to establish baseline voiding frequency, bladder capacity (Figure 4A), and void volume associated with acute (4 h) CYP treatment (data not shown). As previously demonstrated (Vizzard, 2000a,c; Hu et al., 2005; Braas et al., 2006; Cheppudira et al., 2009; Arms et al., 2010), CYP treatment (4 h) increased void frequency and decreased bladder capacity (1.8-fold), intercontraction interval (1.7-fold), and void volume in comparison to control mice (no CYP treatment). Acute CYP-induced cystitis (4 h) also increased the number and amplitude of NVCs (Figure 4B). Following intravesical infusion of the CXCR3 antagonist, AMG487 (5 mg/kg; post 4 h CYP), the same CYP-treated female mice exhibited decreased (*p* ≤ 0.01) voiding frequency (lengthened intercontraction interval; data not shown) and significantly (*p* ≤ 0.01) increased bladder capacity, as measured as the amount of saline infused in the bladder at the time when micturition commenced (Figure 4A), and increased void volume (data not shown). There were no changes in threshold, minimum, average or maximum pressures following intravesical instillation with the CXCR3 antagonist infusion in 4 h CYP-treated rats (Figure 7). CXCR3 blockade at the level of the urinary bladder in acute (4 h) cystitis reduced the number of NVCs (Figure 4B). The amplitude of NVCs significantly (*p* ≤ 0.01) decreased (13.7 ± 3.2 cm H_2_O vs. 6.7 ± 1.4 cm H_2_O) in 48 h CYP treated mice with intravesical CXCR3 receptor antagonist instillation.

#### 48 h CYP Treatment

Conscious cystometry was also performed in female mice treated with CYP treatment (48 h) and vehicle before intravesical infusion of AMG487 (5 mg/kg; pre 48 h CYP) to establish baseline voiding frequency, bladder capacity, and void volume associated with CYP treatment (48 h; Figures 4A, 6A1,A2). Comparable to changes in urinary bladder function with acute (4 h) CYP treatment, intermediate (48 h) cystitis similarly increased void frequency and decreased bladder capacity (1.8-fold), intercontraction interval (1.8-fold), and void volume in comparison to control mice (no CYP treatment; Figures 4A, 6A1,A2). Intermediate (48 h) CYP-induced cystitis also increased the number and amplitude of NVCs (Figures 4B, 6A1,A2). Following intravesical infusion of the CXCR3 antagonist, AMG487 (5 mg/kg; post 48 h CYP), the same CYP-treated female mice exhibited decreased voiding frequency (lengthened intercontraction interval) and significantly (*p* ≤ 0.01) increased bladder capacity (Figures 4A, 6B1,B2), and void volume (data not shown). There were no changes in threshold, minimum, average or maximum pressures following intravesical instillation with the CXCR3 antagonist infusion in 4 h CYP-treated rats (Figure 7). CXCR3 blockade at the level of the urinary bladder in intermediate (48 h) cystitis reduced the number NVCs (Figure 4B). The amplitude of NVCs significantly (*p* ≤ 0.01) decreased (10.2 ± 2.2 cm H_2_O vs. 5.5 ± 0.3 cm H_2_O) in 48 h CYP treated mice with intravesical CXCR3 receptor antagonist instillation.

### Hindpaw and Pelvic Somatic Sensitivity With CYP-Induced Cystitis (4 h) in Female Mice and Effects of CXCR3 Receptor Antagonist, AMG487

**Figure 8:**
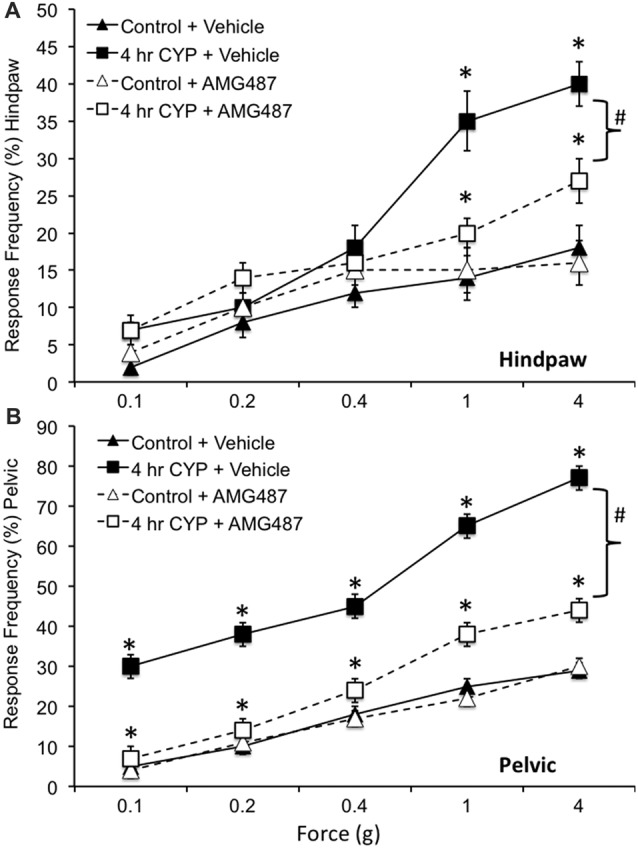
Somatic sensitivity of hindpaw and pelvic region in female mice is increased with CYP treatment and reduced with intravesical instillation of the CXCR3 receptor antagonist, AMG487 (5 mg/kg). Somatic sensitivity testing of the hindpaw **(A)** and pelvic regions **(B)** with von Frey filaments in control (closed triangles) mice with vehicle, mice treated with CYP (4 h; closed squares) and vehicle, CYP treated (4 h) mice with intravesical instillation of a CXCR3 receptor antagonist, AMG487 (5 mg/kg; open squares) and control (open triangles) mice with intravesical infusion of AMG487 (5 mg/kg). The von Frey filaments were applied in an up-down method for 1–3 s with an interstimulus interval of 15 s. A positive response to hindpaw stimulation was sharp withdrawal of the paw or licking of the tested hindpaw. For pelvic region stimulation, stimulation was confined to the lower abdominal area overlying the urinary bladder. The following behaviors were considered positive responses to pelvic region stimulation: sharp retraction of the abdomen, jumping, or immediate licking or scratching of the pelvic area. Mice treated acutely (4 h) with CYP and vehicle had a significantly (*p ≤ 0.01) increased hindpaw response frequency with filaments of greater force (1 g, 4 g) **(A)** and pelvic response frequency **(B)** with all von Frey filaments (0.1–4 g) tested compared to control mice (no CYP + vehicle). Mice treated acutely (4 h) with CYP and intravesical infusion of AMG487 had a significantly (bracket with ^#^*p* ≤ 0.01) reduced hindpaw response frequency with filaments of greater force (1 g, 4 g) **(A)** and pelvic response frequency **(B)** with all von Frey filaments (0.1–4 g) tested compared to CYP (4 h + vehicle). Although somatic sensitivity in the hindpaw and pelvic region were significantly (*p* ≤ 0.01) reduced in female mice with CYP-induced cystitis and AMG487 treatment, this effect was only partial because somatic sensitivity in these mice was still significantly (*p* ≤ 0.01) greater than that in control + AMG487 at greater forces evaluated **(A,B)**. No differences in hindpaw **(A)** or pelvic sensitivity **(B)** were detected between control mice treated with vehicle and control mice treated with AMG487. Hindpaw or pelvic sensitivity testing with calibrated von Frey filaments was determined in separate groups of female mice. All somatic testing was performed in a blinded manner and decoded after testing for analyses. Sample sizes are *n =* 10 for each group; *p ≤ 0.01.

Somatic sensitivity in the hindpaw was significantly (*p* ≤ 0.01) increased with the highest monofilaments forces tested (1 g and 4 g) following 4 h CYP treatment compared to control female mice (Figure 8A) as previously described (Cheppudira et al., 2009). Similarly, pelvic somatic sensitivity was also significantly (*p* ≤ 0.01) increased following 4 h CYP treatment at all monofilament forces evaluated (0.1–4 g; Figure 8B) compared to control female mice as previously described (Arms et al., 2013). Intravesical infusion of AMG487 (5 mg/kg) significantly (*p* ≤ 0.01) decreased the somatic sensitivity in the hindpaw (Figure 8A) and pelvic region (Figure 8B) in female mice with CYP-induced cystitis (4 h). Although somatic sensitivity in the hindpaw and pelvic region were significantly (*p* ≤ 0.01) reduced in female mice with CYP-induced cystitis and AMG487 treatment, this effect was only partial because somatic sensitivity in these mice was still significantly (*p* ≤ 0.01) greater than that in control mice (Figures 8A,B) at greater forces evaluated. Intravesical infusion of the CXCR3 receptor antagonist in control female mice (no CYP treatment) produced no change in somatic sensitivity in the hindpaw (Figure 8A) or pelvic region (Figure 8B).

### CXCR3 Receptor Expression in Urinary Bladder of Female Mice

**Figure 9:**
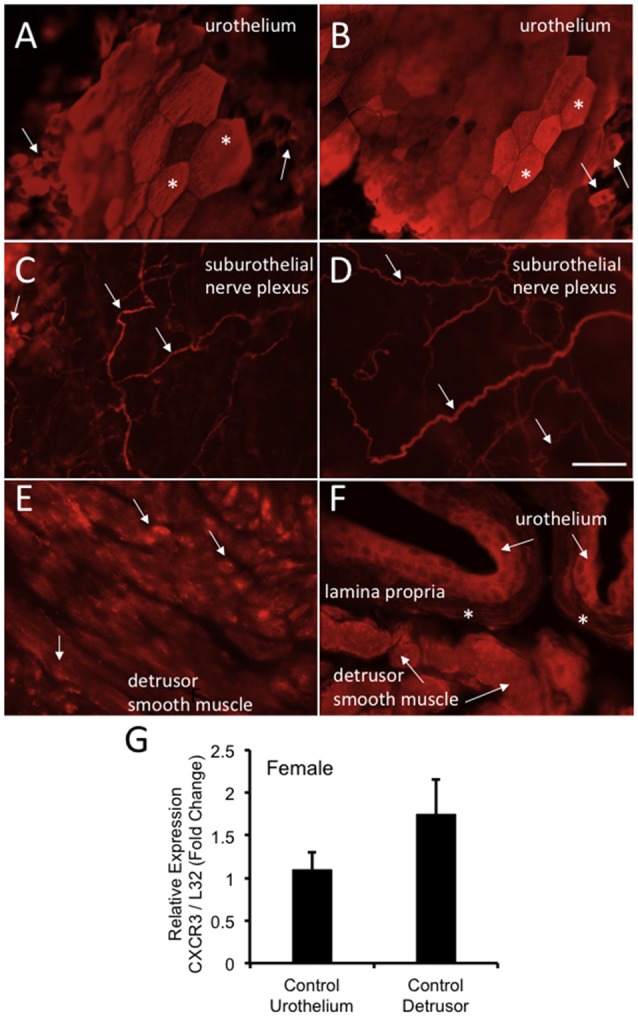
CXCR3-immunoreactivity (IR) in the urinary bladder (wholemounts and cryostat sections) of control female mice. **(A,B)** Epifluorescence images of wholemount urothelium preparations from control mice with robust CXCR3-IR in large, hexagonally shaped umbrella cells (*) and smaller urothelial cells that may represent basal and intermediate populations (arrows). **(C,D)** Epifluorescence images of wholemount urothelium preparations with robust and widespread CXCR3-IR in suburothelial nerve fibers (arrows). **(C,D)** CXCR3-IR was expressed in larger and smaller caliber nerve fibers. **(E)** Epifluorescence image of CXCR3-IR in cryostat bladder sections of the detrusor smooth muscle from control mice. CXCR3-IR in the detrusor smooth muscle was punctate in nature and diffusely distributed (arrows). **(F)** Epifluorescence image of a cryostat section of urinary bladder with CXCR3-IR expression in the urothelium (arrows), the lamina propria (*) and detrusor smooth muscle (arrows). Little CXCR3-IR was present in the lamina propria. Calibration bar represents 80 μm in **(A–D)** and 50 μm in **(E,F)**. **(G)** CXCR3 mRNA transcript expression in the urothelium and detrusor smooth muscle of control female mice as determined by qRT-PCR. CXCR3 mRNA is expressed as the fold change of relative CXCR3 expression/relative L32 expression and normalized to expression in urothelium. Sample sizes are *n* = 3–5 in each group.

CXCR3-IR was robustly expressed in wholemount preparations of the urothelium from control (no CYP) female mice being expressed in large, apical urothelial cells (Figures 9A,B) as well as in smaller, intermediate or basal cells (Figures 9A–C). CXCR3-IR was also extensively observed in wholemount preparations of the suburothelial nerve plexus with both larger and smaller nerve fibers being labeled in control female mice (Figures 9C,D). CXCR3-immunoreactive nerve fibers were co-labeled with the pan neuronal marker (PGP 9.5; data not shown). In cryostat sections of the urinary bladder, CXCR3-IR was observed in multiple layers of the urothelium (Figure 9F), in the lamina propria (Figure 9F) and diffusely in detrusor smooth muscle from control female mice (Figures 9E,F). CXCR3 mRNA transcript expression was present in the urothelium and detrusor smooth muscle from control (no CYP) female mice (Figure 9G).

## Discussion

The present studies extend our current understanding of CXC/CXCR3 receptor expression and regulation in the urinary bladder of mice with urinary bladder inflammation induced by CYP. CYP-induced cystitis (4 h, 48 h, chronic) produced changes in CXC chemokine mRNA transcript expression in the urothelium and detrusor of female and mice. CXC mRNA transcript expression changes in female mice with cystitis were primarily increases in expression in the urothelium and detrusor for CXCL9 and CXCL10. In contrast, CXC mRNA transcript expression changes in male mice with cystitis were primarily decreases in expression (CXCL9-11) in the urothelium but both increases (CXCL10) and decreases (CXCL9, CXCL11) in expression were observed in the detrusor. Although CXC transcript expression was altered in the urinary bladder of male mice, no changes in CXC protein expression in whole urinary bladder were demonstrated. In contrast, CXC (CXCL9, CXCL10) protein expression in whole urinary bladder of female mice was significantly (*p* ≤ 0.01) increased with 4 h CYP treatment followed by significant (*p* ≤ 0.01) decreases in expression with 48 h and chronic CYP treatment. Bladder function and somatic (pelvic and hindpaw) sensitivity testing were focused on female mice. Intravesical infusion of the CXCR3 receptor antagonist, AMG487 (5 mg/kg) significantly (*p* ≤ 0.01) increased bladder capacity (infused volume), interval between void events and void volumes whereas NVCs were significantly (*p* ≤ 0.01) reduced in amplitude and number following CYP treatment (4 h, 48 h). Intravesical infusion of the CXCR3 receptor antagonist, AMG487 (5 mg/kg), significantly (*p* ≤ 0.01) reduced pelvic and hindpaw sensitivity in female mice with CYP-induced cystitis (4 h, 48 h). Protein and mRNA transcript expression of the common receptor, CXCR3, for CXCL9, CXCL10 and CXCL11 was present in the urothelium, suburothelial nerve plexus and detrusor smooth muscle of control female mice. Targeting CXC/CXCR3 signaling at the level of the urinary bladder may be a novel target to improve bladder dysfunction and somatic sensitization following urinary bladder inflammation.

Chemokines, chemotactic cytokines, are important mediators of the innate immune response but also contribute to nociception and inflammatory processes in many organ systems (Rutkowski and DeLeo, 2002; Tanaka et al., 2004; Verge et al., 2004; Lindia et al., 2005; White et al., 2005a,b; Bhangoo et al., 2007). There is a growing literature on the expression and functional contributions of chemokine/receptor signaling in preclinical animal models of urinary bladder inflammation, and clinical studies with BPS/IC and OAB patients (Sakthivel et al., 2008; Tyagi et al., 2012; Gonzalez et al., 2014a; Guan et al., 2015; Furuta et al., 2018). For example, in a model of chemically induced-bladder inflammation, CYP treatment increases the urothelial expression of the following chemokine/receptors: fractalkine (CX3CL1)/CX3CR1 (Yuridullah et al., 2006), CXCL12/CXCR4 (Arms et al., 2010) and monocyte chemoattractant protein-1(CCL2)/CCR2 (Arms et al., 2013). Receptor blockade of CXCR4 at the level of the urinary bladder reduces CYP-induced increases in voiding frequency, bladder capacity and void volume in rats (Arms et al., 2010). Previous studies (Tanaka et al., 2004; White et al., 2005a,b; Dansereau et al., 2008) also demonstrated that CCL2/CCR2 interactions contribute to inflammation-induced bladder dysfunction and increased referred somatic sensitivity. A broader role for CCL2/CCR2 signaling has been demonstrated in nociception following neuronal inflammation or chronic nerve injury in the CNS and PNS (Abbadie et al., 2003; Tanaka et al., 2004; Qin et al., 2005; Menetski et al., 2007; Dansereau et al., 2008; Gao et al., 2009; Van Steenwinckel et al., 2011). Transgenic mouse studies with CCR2 null mice and mice with CCL2 overexpression have also corroborated roles for CCL2/CCR2 signaling in hypersensitivity and hyperalgesia following partial sciatic nerve ligation (Abbadie et al., 2003) or complete Freund’s adjuvant-induced inflammation (Menetski et al., 2007). Tyagi et al. (2012) detected a 10-fold increase of CCL2, the soluble fraction of the CD40 ligand (CD40L), various cytokines, epidermal growth factor and the oncogene GRO-2 were also elevated (3–5-fold).

CXC chemokines are named for their C-X-C amino acid motif in their N-terminus that contains two cysteine residues separated by another amino acid (X; Murphy et al., 2000). The CXC chemokine family is pro-inflammatory with family members CXCL9, CXCL10, CXCL11, and their common receptor, CXCR3, contributing to urinary bladder inflammation (Sakthivel et al., 2008; Guan et al., 2015). Previous studies have shown that anti-CXCL10 treatment reduces CYP-induced symptoms such as urinary bladder leukocyte infiltration, hyperplasia and epithelial erosions (Sakthivel et al., 2008; Guan et al., 2015). In addition, Sakthivel et al. (2008) demonstrated that anti-CXCL10 in female mice decreased production of CXCR3 ligands and local T helper type 1 (Th1) cytokines expressed on leukocytes. BPS/IC patients demonstrate increased serum expression of CXC chemokines and Th1 cytokines (Sakthivel et al., 2008). Anti-CXCL10 treatment in mice with EAC reduced CXCR3 ligand mRNA in bladder sites and normalized the number and percentage of CD4+ T cells in EAC mice (Singh et al., 2013). The current studies extend these reports by: evaluating CXC chemokines in male and female mice with several CYP treatment durations (4 h, 48 h, chronic), evaluating CXCR3 receptor blockade on bladder function and somatic sensitivity in mice with CYP-induced cystitis, and determining CXCR3 receptor expression in the urinary bladder.

The current studies demonstrate increased expression of CXC chemokines (CXCL9, CXCL10) in the urinary bladder with CYP-induced cystitis (4 h) and improved functional changes in the urinary bladder and somatic sensation (pelvic and hindpaw) with CYP-induced cystitis (4 h and 48 h) following CXCR3 receptor blockade in female mice. These studies suggest that CXC/CXCR3 signaling, at the level of the urinary bladder, is another example of the importance of chemokine/receptor signaling in micturition reflexes with urinary bladder inflammation (Yuridullah et al., 2006; Arms et al., 2010, 2013; Gonzalez et al., 2014a). Recent studies suggest that chemokines secreted by their host cells may signal in a paracrine or autocrine manner to facilitate hyperexcitability (Qin et al., 2005; White et al., 2005a,b; Bhangoo et al., 2007; Gao et al., 2009, 2010) by changing membrane potentials, decreasing thresholds for action potential generation, modulating calcium ion currents and increasing excitability and evoking discharges (Qin et al., 2005; White et al., 2005a,b; Bhangoo et al., 2007; Gao et al., 2009, 2010). In either paradigm, the expression of CXCR3 on urothelial cells, suburothelial nerves and detrusor smooth muscle provides a potential mechanism for CXC/CXCR3 signaling at the level of the urinary bladder to induce functional sensory and motor changes. The reductions in voiding frequency, bladder capacity and non-voiding contractions (NVCs) achieved with intravesical instillation of the CXCR3 receptor antagonist, AMG487, are consistent with effects on the sensory limb of the micturition reflex (e.g., frequency, capacity) as well as the urinary bladder (e.g., NVCs) although the underlying mechanisms of NVCs are still being determined (Heppner et al., 2016; Drake et al., 2017). In pilot studies to determine an effective concentration for intravesical delivery of AMG487, we evaluated concentrations of AMG487 greater than that used in the full study. Intravesical concentrations of AMG487 greater than 5 mg/kg, did not produce greater, beneficial effects in bladder function parameters in CYP-treated mice. In the current study, intravesical AMG487 (5 mg/kg) produced comparable effects in bladder function parameters in CYP-treated (4 h, 48 h) mice.

Urothelial cells share a number of similarities with sensory neurons, including the expression of receptors including purinergic, norepinephrine, acetylcholine, neuropeptide and protease-activated and, neurotrophin receptors as well as ion channels including acid sensing ion channels, and transient receptor potential channels (Sun et al., 2001; Sun and Chai, 2004, 2006; Birder, 2005, 2007; Apodaca et al., 2007; Birder and Andersson, 2013; Merrill et al., 2013; Gonzalez et al., 2014a,b; Girard et al., 2017). In the current study, CXCR3 expression was robustly and widely expressed in the urothelium and suburothelial nerves. CXCR3 expression in the suburothelial nerve plexus distinguishes this receptor from other chemokine receptors (CX3CR1, CXCR4, CCR2) examined in our lab (Yuridullah et al., 2006; Arms et al., 2010, 2013) that failed to demonstrate expression, which may suggest broader roles for CXC/CXCR3 signaling in the urinary bladder. The current studies do not differentiate between direct urothelial and nerve-mediated CXCR3 effects vs. indirect urothelial-mediated communication with other bladder tissues (e.g., detrusor smooth muscle, suburothelial nerve plexus and/or interstitial cells) as previously suggested (Apodaca et al., 2007; Birder and Andersson, 2013). Whether urothelial CXC/CXCR3 signaling facilitates the release of urothelial-derived mediators such as ATP to influence underlying structures such as the suburothelial nerve plexus and/or detrusor smooth remains to be determined. Future studies should address CXCR3 receptor plasticity in the urinary bladder with CYP-induced cystitis. Our current results only demonstrate CXCR3 receptor expression urothelial cells, detrusor smooth muscle and suburothelial nerve plexus in control female mice but changes in expression with cystitis have not yet been addressed. In addition to examining CXCR3 receptor expression in the urinary bladder with cystitis, both male and female mice should be evaluated in future studies. Although CXC chemokines did not demonstrate regulation in the urinary bladder with cystitis in male mice and male mice were not evaluated in the current functional studies, regulation at the level of the CXCR3 receptor with cystitis may be present and affect CXC/CXCR3 signaling in micturition pathways in the absence of changes in CXC chemokine expression in male mice.

With the growing recognizing of the potential for the role of sex in explaining phenotypic variation (Karp et al., 2017), we used both male and female C57BL/6 mice in the current study. CXC protein regulation was not observed in male mice with CYP-induced cystitis; however, CXC protein regulation was observed in female mice with CYP-induced cystitis. These results were very surprising and unexpected. The immunoassays were repeated with separate groups of male mice and both male and female samples were assayed simultaneously on the same multiwell plates. The CYP concentration and dosing schedule protocols have been used extensively by our laboratory and others and cause consistent and reproducible urinary bladder inflammation characterized by infiltration of immune cells, mucosal erosion and increased bladder mass (Girard et al., 2017). In addition, bladder function studies in male mice treated with CYP (4 h, 48 h) revealed changes in urinary bladder function identical to those observed with female mice treated with CYP (4 h, 48 h; data not shown). Thus, these studies suggest that CYP treatment in male mice is not associated with CXC protein regulation or does not result in an overall effect on CXC protein expression but does produce a variety of changes in CXC transcript expression in the urothelium and detrusor. These data suggest that CXC/CXCR3 signaling in the urinary bladder in the context of CYP-induced cystitis in males and females may be differentially regulated. CXCL9, CXCL10 and CXCL11 share common transcription factors including NF-κB, STAT1 and IFN regulatory factor (IRF)-1 and each chemokine also has unique transcription factors (Kanda et al., 2007; Tyagi et al., 2012; Guan et al., 2015). However, it is not known how the transcription of CXC chemokines is regulated at the level of the urinary bladder in the context of CYP-induced cystitis.

Previous studies have not reported a differential response in chemokine/receptor expression in micturition reflex studies but few studies including those from the Vizzard lab, have examined both sexes and most have focused on female rodents because of the female predominance of BPS/IC (Sakthivel et al., 2008; Singh et al., 2013; Girard et al., 2017). In addition, previous studies examining CXC expression in the context of urinary bladder inflammation have used different mouse strains compared to the C57BL/6 strain used in the present study (Sakthivel et al., 2008; Singh et al., 2013). In the current studies, we did not control for the estrous cycle status of the female mice. Despite this, robust changes in CXC transcript and protein expression were demonstrated in urinary bladder with CYP-induced cystitis. In addition, CXCR3 blockade was effective in reducing urinary frequency, and the appearance and magnitude of NVCs in female mice treated with CYP (4 h and 48 h). Although it is possible that the magnitude of changes could be increased if the estrous cycle status was controlled, variations in estrous cycle status did not limit our ability to detect regulation in CXC/receptor signaling in micturition reflex pathways in CYP-treated female mice. In the present studies, immunoassays for CXC chemokines demonstrated a significant increase in CXC chemokine expression with 4 h CYP treatment but a significant decrease in CXC chemokine expression with 48 h CYP treatment in whole urinary bladder of female mice. CXCR3 blockade at the level of the urinary bladder was effective in improving overall urinary bladder function in female mice with 4 h or 48 h CYP treatment. The reasons underlying the beneficial effect of CXCR3 blockade at the 48 h time point with a decrease in CXC chemokine expression at the 48 h time point are not obvious but may due, in part, to the use of whole urinary bladder for immunoassays rather than isolated urothelium or detrusor tissue. The changes in the protein expression in immunoassays from whole urinary bladder reflect those in the detrusor muscle because it represents the largest tissue of the urinary bladder. If immunoassays of isolated urothelium or detrusor smooth muscle were performed, a different expression pattern for CXC chemokines, particularly in the urothelium, may have been observed. Preparations of isolated mouse urothelium were not used because the protein yield is inadequate requiring pooling of specimens making statistical comparisons challenging. Given the beneficial effects of CXCR3 blockade in female mice with 48 h CYP treatment, future studies should also evaluate effects following chronic CYP treatment.

Blockade of chemokine/receptor signaling may represent a potential therapeutic target for inflammation-associated bladder dysfunction (Tyagi et al., 2012; Gonzalez et al., 2014a; Furuta et al., 2018). The heterogeneity of BPS/IC may underlie why few, effective treatments exist for the successful management of BPS/IC (Andersson and Birder, 2017). Thus, there is a clear need to identify novel targets for therapeutic intervention. The presence of certain chemokines and other inflammatory molecules in patient urine may prove to be useful biomarkers for BPS/IC or OAB (Tyagi et al., 2012; Gonzalez et al., 2014a; Furuta et al., 2018). Whether certain chemokine/receptor interactions and downstream pathways represent non-specific markers of a generalized inflammatory urinary bladder condition or may represent a unique marker of a urinary pelvic pain syndrome remains to be determined. Identification of biomarkers in BPS/IC, OAB or other bladder dysfunction would improve diagnostic strategies, reduce invasiveness to the patient, reduce time to diagnosis, improve exclusionary criteria and guide patient selection for clinical trials.

## Author Contributions

MG, SMM, PC, EH, KT, JO, BMG and MAV: conceived, discussed and outlined the experimental design and analyzed the data. MG, SMM, PC, EH, KT, JO, BMG and HH: performed experiments. MG, KT, JO, PC, BMG and MAV: drafted and revised the article.

## Conflict of Interest Statement

The authors declare that the research was conducted in the absence of any commercial or financial relationships that could be construed as a potential conflict of interest.

## References

[B1] Abbadie C. Lindia J. A. Cumiskey A. M. Peterson L. B. Mudgett J. S. Bayne E. K.. (2003). Impaired neuropathic pain responses in mice lacking the chemokine receptor CCR2. Proc. Natl. Acad. Sci. U S A 100, 7947–7952. 10.1073/pnas.1331358100 12808141 PMC164693

[B2] Andersson K. E. Birder L. (2017). Current pharmacologic approaches in painful bladder research: an update. Int. Neurourol. J. 21, 235–242. 10.5213/inj.1735022.511 29298474 PMC5756823

[B3] Apodaca G. Balestreire E. Birder L. A. (2007). The uroepithelial-associated sensory web. Kidney Int. 72, 1057–1064. 10.1038/sj.ki.5002439 17667988

[B5] Arms L. Girard B. M. Malley S. E. Vizzard M. A. (2013). Expression and function of CCL2/CCR2 in rat micturition reflexes and somatic sensitivity with urinary bladder inflammation. Am. J. Physiol. Renal Physiol. 305, F111–F122. 10.1152/ajprenal.00139.2013 23594826 PMC3725675

[B4] Arms L. Girard B. M. Vizzard M. A. (2010). Expression and function of CXCL12/CXCR4 in rat urinary bladder with cyclophosphamide-induced cystitis. Am. J. Physiol. Renal Physiol. 298, F589–F600. 10.1152/ajprenal.00628.2009 20032115 PMC2838600

[B6] Berry S. H. Elliott M. N. Suttorp M. Bogart L. M. Stoto M. A. Eggers P.. (2011). Prevalence of symptoms of bladder pain syndrome/interstitial cystitis among adult females in the United States. J. Urol. 186, 540–544. 10.1016/j.juro.2011.03.132 21683389 PMC3513327

[B7] Bhangoo S. K. Ren D. Miller R. J. Chan D. M. Ripsch M. S. Weiss C.. (2007). CXCR4 chemokine receptor signaling mediates pain hypersensitivity in association with antiretroviral toxic neuropathy. Brain Behav. Immun. 21, 581–591. 10.1016/j.bbi.2006.12.003 17292584 PMC2062574

[B9] Birder L. A. (2005). More than just a barrier: urothelium as a drug target for urinary bladder pain. Am. J. Physiol. Renal Physiol. 289, F489–F495. 10.1152/ajprenal.00467.2004 16093424

[B10] Birder L. A. (2007). TRPs in bladder diseases. Biochim. Biophys. Acta 1772, 879–884. 10.1016/j.bbadis.2007.04.003 17560087 PMC3713460

[B8] Birder L. Andersson K. E. (2013). Urothelial signaling. Physiol. Rev. 93, 653–680. 10.1152/physrev.00030.2012 23589830 PMC3768101

[B11] Braas K. M. May V. Zvara P. Nausch B. Kliment J. Dunleavy J. D.. (2006). Role for pituitary adenylate cyclase activating polypeptide in cystitis-induced plasticity of micturition reflexes. Am. J. Physiol. Regul. Integr. Comp. Physiol. 290, R951–R962. 10.1152/ajpregu.00734.2005 16322346 PMC1402357

[B12] Cheppudira B. P. Girard B. M. Malley S. E. Dattilio A. Schutz K. C. May V.. (2009). Involvement of JAK-STAT signaling/function after cyclophosphamide-induced bladder inflammation in female rats. Am. J. Physiol. Renal Physiol. 297, F1038–F1044. 10.1152/ajprenal.00110.2009 19625377 PMC2775567

[B13] Clemens J. Q. Mullins C. Kusek J. W. Kirkali Z. Mayer E. A. Rodriguez L. V.. (2014). The MAPP research network: a novel study of urologic chronic pelvic pain syndromes. BMC Urol. 14:57. 10.1186/1471-2490-14-57 25085007 PMC4134515

[B14] Compérat E. Reitz A. Delcourt A. Capron F. Denys P. Chartier-Kastler E. (2006). Histologic features in the urinary bladder wall affected from neurogenic overactivity—a comparison of inflammation, oedema and fibrosis with and without injection of botulinum toxin type A. Eur. Urol. 50, 1058–1064. 10.1016/j.eururo.2006.01.025 16517054

[B15] Corrow K. Girard B. M. Vizzard M. A. (2010). Expression and response of acid-sensing ion channels in urinary bladder to cyclophosphamide-induced cystitis. Am. J. Physiol. Renal Physiol. 298, F1130–F1139. 10.1152/ajprenal.00618.2009 20164155 PMC2867414

[B16] Dansereau M. A. Gosselin R. D. Pohl M. Pommier B. Mechighel P. Mauborgne A.. (2008). Spinal CCL2 pronociceptive action is no longer effective in CCR2 receptor antagonist-treated rats. J. Neurochem. 106, 757–769. 10.1111/j.1471-4159.2008.05429.x 18419759

[B17] Drake M. J. Kanai A. Bijos D. A. Ikeda Y. Zabbarova I. Vahabi B.. (2017). The potential role of unregulated autonomous bladder micromotions in urinary storage and voiding dysfunction; overactive bladder and detrusor underactivity. BJU Int. 119, 22–29. 10.1111/bju.13598 27444952 PMC5177525

[B18] Driscoll A. Teichman J. M. (2001). How do patients with interstitial cystitis present? J. Urol. 166, 2118–2120. 10.1097/00005392-200112000-00023 11696718

[B19] Furuta A. Yamamoto T. Suzuki Y. Gotoh M. Egawa S. Yoshimura N. (2018). Comparison of inflammatory urine markers in patients with interstitial cystitis and overactive bladder. Int. Urogynecol. J. [Epub ahead of print]. 10.1007/s00192-017-3547-5 29372285

[B20] Gao Y. J. Xu Z. Z. Liu Y. C. Wen Y. R. Decosterd I. Ji R. R. (2010). The c-Jun N-terminal kinase 1 (JNK1) in spinal astrocytes is required for the maintenance of bilateral mechanical allodynia under a persistent inflammatory pain condition. Pain 148, 309–319. 10.1016/j.pain.2009.11.017 20022176 PMC2814908

[B21] Gao Y. J. Zhang L. Samad O. A. Suter M. R. Yasuhiko K. Xu Z. Z.. (2009). JNK-induced MCP-1 production in spinal cord astrocytes contributes to central sensitization and neuropathic pain. J. Neurosci. 29, 4096–4108. 10.1523/jneurosci.3623-08.2009 19339605 PMC2682921

[B24] Girard B. M. Malley S. E. Braas K. M. May V. Vizzard M. A. (2010). PACAP/VIP and receptor characterization in micturition pathways in mice with overexpression of NGF in urothelium. J. Mol. Neurosci. 42, 378–389. 10.1007/s12031-010-9384-3 20449688 PMC2955834

[B25] Girard B. M. Malley S. E. Braas K. M. Waschek J. A. May V. Vizzard M. A. (2008). Exaggerated expression of inflammatory mediators in vasoactive intestinal polypeptide knockout (VIP^−/–^) mice with cyclophosphamide (CYP)-induced cystitis. J. Mol. Neurosci. 36, 188–199. 10.1007/s12031-008-9084-4 18483878 PMC2695563

[B23] Girard B. M. Malley S. E. Vizzard M. A. (2011). Neurotrophin/receptor expression in urinary bladder of mice with overexpression of NGF in urothelium. Am. J. Physiol. Renal Physiol. 300, F345–F355. 10.1152/ajprenal.00515.2010 21048026 PMC3043996

[B26] Girard B. M. Merrill L. Malley S. Vizzard M. A. (2013). Increased TRPV4 expression in urinary bladder and lumbosacral dorsal root ganglia in mice with chronic overexpression of NGF in urothelium. J. Mol. Neurosci. 51, 602–614. 10.1007/s12031-013-0033-5 23690258 PMC3779511

[B22] Girard B. Peterson A. Malley S. Vizzard M. A. (2016). Accelerated onset of the vesicovesical reflex in postnatal NGF-OE mice and the role of neuropeptides. Exp. Neurol. 285, 110–125. 10.1016/j.expneurol.2016.06.021 27342083 PMC5077658

[B27] Girard B. M. Tooke K. Vizzard M. A. (2017). PACAP/receptor system in urinary bladder dysfunction and pelvic pain following urinary bladder inflammation or stress. Front. Syst. Neurosci. 11:90. 10.3389/fnsys.2017.00090 29255407 PMC5722809

[B28] Gonzalez E. J. Arms L. Vizzard M. A. (2014a). The role(s) of cytokines/chemokines in urinary bladder inflammation and dysfunction. Biomed Res. Int. 2014:120525. 10.1155/2014/120525 24738044 PMC3971501

[B30] Gonzalez E. J. Merrill L. Vizzard M. A. (2014b). Bladder sensory physiology: neuroactive compounds and receptors, sensory transducers and target-derived growth factors as targets to improve function. Am. J. Physiol. Regul. Integr. Comp. Physiol. 306, R869–R878. 10.1152/ajpregu.00030.2014 24760999 PMC4159737

[B29] Gonzalez E. J. Girard B. M. Vizzard M. A. (2013). Expression and function of transforming growth factor-beta isoforms and cognate receptors in the rat urinary bladder following cyclophosphamide-induced cystitis. Am. J. Physiol. Renal Physiol. 305, F1265–F1276. 10.1152/ajprenal.00042.2013 23926183 PMC3840223

[B31] Gonzalez E. J. Peterson A. Malley S. Daniel M. Lambert D. Kosofsky M.. (2015). The effects of tempol on cyclophosphamide-induced oxidative stress in rat micturition reflexes. ScientificWorldJournal 2015:545048. 10.1155/2015/545048 25973443 PMC4417973

[B32] Gosselin R. D. Dansereau M. A. Pohl M. Kitabgi P. Beaudet N. Sarret P.. (2008). Chemokine network in the nervous system: a new target for pain relief. Curr. Med. Chem. 15, 2866–2875. 10.2174/092986708786242822 18991641

[B33] Guan X. H. Fu Q. C. Shi D. Bu H. L. Song Z. P. Xiong B. R.. (2015). Activation of spinal chemokine receptor CXCR3 mediates bone cancer pain through an Akt-ERK crosstalk pathway in rats. Exp. Neurol. 263, 39–49. 10.1016/j.expneurol.2014.09.019 25281485

[B34] Guerios S. D. Wang Z. Y. Boldon K. Bushman W. Bjorling D. E. (2008). Blockade of NGF and trk receptors inhibits increased peripheral mechanical sensitivity accompanying cystitis in rats. Am. J. Physiol. Regul. Integr. Comp. Physiol. 295, R111–R122. 10.1152/ajpregu.00728.2007 18448607 PMC2494812

[B35] Hanno P. M. Sant G. R. (2001). Clinical highlights of the national institute of diabetes and digestive and kidney diseases/interstitial cystitis association scientific conference on interstitial cystitis. Urology 57, 2–6. 10.1016/s0090-4295(01)01112-8 11378041

[B36] Heppner T. J. Tykocki N. R. Hill-Eubanks D. Nelson M. T. (2016). Transient contractions of urinary bladder smooth muscle are drivers of afferent nerve activity during filling. J. Gen. Physiol. 147, 323–335. 10.1085/jgp.201511550 26976828 PMC4810069

[B37] Hu V. Y. Zvara P. Dattilio A. Redman T. L. Allen S. J. Dawbarn D.. (2005). Decrease in bladder overactivity with REN1820 in rats with cyclophosphamide induced cystitis. J. Urol. 173, 1016–1021. 10.1097/01.ju.0000155170.15023.e5 15711368

[B38] Kanda N. Shimizu T. Tada Y. Watanabe S. (2007). IL-18 enhances IFN-gamma-induced production of CXCL9, CXCL10 and CXCL11 in human keratinocytes. Eur. J. Immunol. 37, 338–350. 10.1002/eji.200636420 17274000

[B39] Karp N. A. Heller R. Yaacoby S. White J. K. Benjamini Y. (2017). Improving the identification of phenotypic abnormalities and sexual dimorphism in mice when studying rare event categorical characteristics. Genetics 205, 491–501. 10.1534/genetics.116.195388 27932544 PMC5289831

[B40] Lindia J. A. McGowan E. Jochnowitz N. Abbadie C. (2005). Induction of CX3CL1 expression in astrocytes and CX3CR1 in microglia in the spinal cord of a rat model of neuropathic pain. J. Pain 6, 434–438. 10.1016/j.jpain.2005.02.001 15993821

[B41] Malley S. E. Vizzard M. A. (2002). Changes in urinary bladder cytokine mRNA and protein after cyclophosphamide-induced cystitis. Physiol. Genomics 9, 5–13. 10.1152/physiolgenomics.00117.2001 11948286

[B42] Mehrad B. Keane M. P. Strieter R. M. (2007). Chemokines as mediators of angiogenesis. Thromb. Haemost. 97, 755–762. 10.1160/th07-01-0040 17479186 PMC3353527

[B43] Menetski J. Mistry S. Lu M. Mudgett J. S. Ransohoff R. M. Demartino J. A.. (2007). Mice overexpressing chemokine ligand 2 (CCL2) in astrocytes display enhanced nociceptive responses. Neuroscience 149, 706–714. 10.1016/j.neuroscience.2007.08.014 17870246

[B44] Merrill L. Girard B. Arms L. Guertin P. Vizzard M. A. (2013). Neuropeptide/Receptor expression and plasticity in micturition pathways. Curr. Pharm. Des. 19, 4411–4422. 10.2174/1381612811319240008 23360273

[B45] Milligan E. D. Sloane E. M. Watkins L. R. (2008). Glia in pathological pain: a role for fractalkine. J. Neuroimmunol. 198, 113–120. 10.1016/j.jneuroim.2008.04.011 18547654 PMC3289257

[B46] Murphy P. M. Baggiolini M. Charo I. F. Hebert C. A. Horuk R. Matsushima K.. (2000). International union of pharmacology. XXII. Nomenclature for chemokine receptors. Pharmacol. Rev. 52, 145–176. 10699158

[B47] Murphy S. F. Schaeffer A. J. Thumbikat P. (2014). Immune mediators of chronic pelvic pain syndrome. Nat. Rev. Urol. 11, 259–269. 10.1038/nrurol.2014.63 24686526 PMC4986688

[B48] Ogawa T. Homma T. Igawa Y. Seki S. Ishizuka O. Imamura T.. (2010). CXCR3 binding chemokine and TNFSF14 over expression in bladder urothelium of patients with ulcerative interstitial cystitis. J. Urol. 183, 1206–1212. 10.1016/j.juro.2009.11.007 20096889

[B49] Parsons C. L. (2007). The role of the urinary epithelium in the pathogenesis of interstitial cystitis/prostatitis/urethritis. Urology 69, 9–16. 10.1016/j.urology.2006.03.084 17462486

[B50] Patnaik S. S. Laganà A. S. Vitale S. G. Butticè S. Noventa M. Gizzo S.. (2017). Etiology, pathophysiology and biomarkers of interstitial cystitis/painful bladder syndrome. Arch. Gynecol. Obstet. 295, 1341–1359. 10.1007/s00404-017-4364-2 28391486

[B51] Pillalamarri N. Shalom D. F. Pilkinton M. L. Winkler H. A. Chatterjee P. K. Solanki M.. (2017). Inflammatory urinary cytokine expression and quality of life in patients with overactive bladder. Female Pelvic Med. Reconstr. Surg. [Epub ahead of print]. 10.1097/spv.0000000000000492 28953078

[B52] Qin X. Wan Y. Wang X. (2005). CCL2 and CXCL1 trigger calcitonin gene-related peptide release by exciting primary nociceptive neurons. J. Neurosci. Res. 82, 51–62. 10.1002/jnr.20612 16047385

[B53] Rutkowski M. D. DeLeo J. A. (2002). The role of cytokines in the initiation and maintenance of chronic pain. Drug News Perspect. 15, 626–632. 10.1358/dnp.2002.15.10.740239 12677247

[B54] Sakthivel S. K. Singh U. P. Singh S. Taub D. D. Novakovic K. R. Lillard J. W. Jr. (2008). CXCL10 blockade protects mice from cyclophosphamide-induced cystitis. J. Immune Based Ther. Vaccines 6:6. 10.1186/1476-8518-6-6 18957084 PMC2583981

[B55] Sant G. R. Hanno P. M. (2001). Interstitial cystitis: current issues and controversies in diagnosis. Urology 57, 82–88. 10.1016/s0090-4295(01)01131-1 11378054

[B56] Savarin-Vuaillat C. Ransohoff R. M. (2007). Chemokines and chemokine receptors in neurological disease: raise, retain, or reduce? Neurotherapeutics 4, 590–601. 10.1016/j.nurt.2007.07.004 17920540 PMC7479679

[B57] Schnegelsberg B. Sun T. T. Cain G. Bhattacharya A. Nunn P. A. Ford A. P.. (2010). Overexpression of NGF in mouse urothelium leads to neuronal hyperinnervation, pelvic sensitivity and changes in urinary bladder function. Am. J. Physiol. Regul. Integr. Comp. Physiol. 298, R534–R547. 10.1152/ajpregu.00367.2009 20032263 PMC2838659

[B58] Singh U. P. Singh N. P. Guan H. Hegde V. L. Price R. L. Taub D. D.. (2013). The severity of experimental autoimmune cystitis can be ameliorated by anti-CXCL10 Ab treatment. PLoS One 8:e79751. 10.1371/journal.pone.0079751 24278169 PMC3836899

[B59] Steers W. (2000). Pathogenesis of the overactive bladder and its attendant risk factors. BJU Int. 85:69; discussion 70–61. 10.1111/j.1464-410x.2000.tb16959.x 11954201

[B60] Studeny S. Cheppudira B. P. Meyers S. Balestreire E. M. Apodaca G. Birder L. A.. (2008). Urinary bladder function and somatic sensitivity in vasoactive intestinal polypeptide (VIP)^−/–^ mice. J. Mol. Neurosci. 36, 175–187. 10.1007/s12031-008-9100-8 18561033 PMC2693375

[B61] Sun Y. Chai T. C. (2004). Up-regulation of P2X3 receptor during stretch of bladder urothelial cells from patients with interstitial cystitis. J. Urol. 171, 448–452. 10.1097/01.ju.0000099660.46774.3c 14665953

[B62] Sun Y. Chai T. C. (2006). Augmented extracellular ATP signaling in bladder urothelial cells from patients with interstitial cystitis. Am. J. Physiol. Cell Physiol. 290, C27–C34. 10.1152/ajpcell.00552.2004 16107499

[B63] Sun Y. Keay S. De Deyne P. G. Chai T. C. (2001). Augmented stretch activated adenosine triphosphate release from bladder uroepithelial cells in patients with interstitial cystitis. J. Urol. 166, 1951–1956. 10.1097/00005392-200111000-00089 11586266

[B64] Tanaka T. Minami M. Nakagawa T. Satoh M. (2004). Enhanced production of monocyte chemoattractant protein-1 in the dorsal root ganglia in a rat model of neuropathic pain: possible involvement in the development of neuropathic pain. Neurosci. Res. 48, 463–469. 10.1016/j.neures.2004.01.004 15041200

[B65] Tyagi P. Killinger K. Tyagi V. Nirmal J. Chancellor M. Peters K. M. (2012). Urinary chemokines as noninvasive predictors of ulcerative interstitial cystitis. J. Urol. 187, 2243–2248. 10.1016/j.juro.2012.01.034 22503040 PMC3674640

[B66] Van Steenwinckel J. Reaux-Le Goazigo A. Pommier B. Mauborgne A. Dansereau M. A. Kitabgi P.. (2011). CCL2 released from neuronal synaptic vesicles in the spinal cord is a major mediator of local inflammation and pain after peripheral nerve injury. J. Neurosci. 31, 5865–5875. 10.1523/jneurosci.5986-10.2011 21490228 PMC6622829

[B67] Vera P. L. Iczkowski K. A. Wang X. Meyer-Siegler K. L. (2008). Cyclophosphamide-induced cystitis increases bladder CXCR4 expression and CXCR4-macrophage migration inhibitory factor association. PLoS One 3:e3898. 10.1371/journal.pone.0003898 19066630 PMC2588654

[B68] Verge G. M. Milligan E. D. Maier S. F. Watkins L. R. Naeve G. S. Foster A. C. (2004). Fractalkine (CX3CL1) and fractalkine receptor (CX3CR1) distribution in spinal cord and dorsal root ganglia under basal and neuropathic pain conditions. Eur. J. Neurosci. 20, 1150–1160. 10.1111/j.1460-9568.2004.03593.x 15341587

[B69] Vizzard M. A. (2000a). Alterations in spinal cord Fos protein expression induced by bladder stimulation following cystitis. Am. J. Physiol. Regul. Integr. Comp. Physiol. 278, R1027–R1039. 10.1152/ajpregu.2000.278.4.r1027 10749792

[B70] Vizzard M. A. (2000b). Changes in urinary bladder neurotrophic factor mRNA and NGF protein following urinary bladder dysfunction. Exp. Neurol. 161, 273–284. 10.1006/exnr.1999.7254 10683293

[B71] Vizzard M. A. (2000c). Up-regulation of pituitary adenylate cyclase-activating polypeptide in urinary bladder pathways after chronic cystitis. J. Comp. Neurol. 420, 335–348. 10.1002/(SICI)1096-9861(20000508)420:3<335::AID-CNE5>3.0.CO;2-# 10754506

[B72] Vizzard M. A. (2001). Alterations in neuropeptide expression in lumbosacral bladder pathways following chronic cystitis. J. Chem. Neuroanat. 21, 125–138. 10.1016/s0891-0618(00)00115-0 11312054

[B73] Vizzard M. A. (2006). Neurochemical plasticity and the role of neurotrophic factors in bladder reflex pathways after spinal cord injury. Prog. Brain Res. 152, 97–115. 10.1016/s0079-6123(05)52007-7 16198696

[B74] Walser T. C. Rifat S. Ma X. Kundu N. Ward C. Goloubeva O.. (2006). Antagonism of CXCR3 inhibits lung metastasis in a murine model of metastatic breast cancer. Cancer Res. 66, 7701–7707. 10.1158/0008-5472.can-06-0709 16885372

[B75] Watkins L. R. Milligan E. D. Maier S. F. (2003). Glial proinflammatory cytokines mediate exaggerated pain states: implications for clinical pain. Adv. Exp. Med. Biol. 521, 1–21. 12617561

[B76] White F. A. Bhangoo S. K. Miller R. J. (2005a). Chemokines: integrators of pain and inflammation. Nat. Rev. Drug Discov. 4, 834–844. 10.1038/nrd1852 16224455 PMC2792904

[B77] White F. A. Sun J. Waters S. M. Ma C. Ren D. Ripsch M.. (2005b). Excitatory monocyte chemoattractant protein-1 signaling is up-regulated in sensory neurons after chronic compression of the dorsal root ganglion. Proc. Natl. Acad. Sci. U S A 102, 14092–14097. 10.1073/pnas.0503496102 16174730 PMC1236537

[B79] Yoshimura N. Bennett N. E. Hayashi Y. Ogawa T. Nishizawa O. Chancellor M. B.. (2006). Bladder overactivity and hyperexcitability of bladder afferent neurons after intrathecal delivery of nerve growth factor in rats. J. Neurosci. 26, 10847–10855. 10.1523/jneurosci.3023-06.2006 17050722 PMC6674760

[B78] Yoshimura N. de Groat W. C. (1999). Increased excitability of afferent neurons innervating rat urinary bladder after chronic bladder inflammation. J. Neurosci. 19, 4644–4653. 10341262 10.1523/JNEUROSCI.19-11-04644.1999 PMC6782608

[B80] Yoshimura N. Seki S. Chancellor M. B. de Groat W. C. Ueda T. (2002). Targeting afferent hyperexcitability for therapy of the painful bladder syndrome. Urology 59, 61–67. 10.1016/s0090-4295(01)01639-9 12007524

[B81] Yuridullah R. Corrow K. A. Malley S. E. Vizzard M. A. (2006). Expression of fractalkine and fractalkine receptor in urinary bladder after cyclophosphamide (CYP)-induced cystitis. Auton Neurosci. 126–127, 380–389. 10.1016/j.autneu.2006.02.030 16651033 PMC1475778

[B82] Zvara P. Vizzard M. A. (2007). Exogenous overexpression of nerve growth factor in the urinary bladder produces bladder overactivity and altered micturition circuitry in the lumbosacral spinal cord. BMC Physiol. 7:9. 10.1186/1472-6793-7-9 17725832 PMC2000875

